# Supplementary material for: Feature-specific environmental contributions to human cortical architecture revealed by twin brainprints
Source: Commun Biol. 2026 Jul 20;9:1503. doi: 10.1038/s42003-026-10707-w (PMC13752308; doi:10.1038/s42003-026-10707-w)
Supplement: Supplementary file 1 [file 42003_2026_10707_MOESM1_ESM.pdf]

nature portfolio

Peer Review File

Feature-specific environmental contributions to human cortical architecture revealed by twin brainprints

Corresponding Author: Professor Kristine Walhovd

This file contains all reviewer reports in order by version, followed by all author rebuttals in order by version.

Version 0:

Reviewer comments:

Reviewer #1

(Remarks to the Author)
In their manuscript, Walhovd et al. investigated a brain similarity index, brainprint, in a large sample of monozygotic (identical) and dizygotic (fraternal) twins at baseline and after exposure to intensive training in a virtual reality (VR)-based urban environment. They show that brainprints of monozygotic twins that differed in their birth weight, indicating differences in the prenatal environment, were less similar than those of their counterparts with more similar birth weights. Moreover, after the 10-week VR-intervention, they observed changes in brain curvature, showing training-induced plasticity, whereby monozygotic twins showed greater similarity after training, and the brainprints of dizygotic twins differed even more due to the intervention. This manuscript contributes to our understanding of how genetic influences and environmental factors interact to affect certain aspects of cortical brain features.
The study and analyses are extensive and well described. I would like to commend the authors for their innovative and demanding work, not least the ingenuity with which they named the VR-based city and designed its layout. Overall, this work and manuscript are of high quality. I only have a few minor comments that the authors may wish to consider.

Main comment
When calculating brainprints, are the area and volumetric features normalised for total intracranial volume? Given the distributions of the individual features (Figure 2D and Figure 4), it seems to me that the ICV may have a slight influence on the area, surface and curvature (as expected, not on the thickness). Even for the curvature, although relatively safe, nonlinear allometric scaling can still subtly alter the pattern of folding. Although MZ are safe, DZ might reflect larger variability.

Minor comments
Abstract/Introduction
1. You introduce the term brainprint quite late; perhaps consider adding a general short explanation already in the abstract. (Up until the Methods section, I was wondering how it differs from or is connected to the BrainPrint.)
(2.) p. 4, line 72: 'We examine how human cortical architecture – surface area, thickness, volume, and curvature holistically and feature-wise are related to different environmental factors and interrogate effects further using measures of cortical microstructure.' I find this sentence a little awkward. Perhaps consider rewriting it.
(3.) p. 7, line 149 Perhaps consider rewriting this sentence: 'we included for analyses a series of lifestyle-related variables.'
Methods
(4.) What was the sample composition gender/sex-wise?
(5.) It would be interesting to see the associations with the cognitive/performance scores from the training, but I can imagine this is beyond the scope of this manuscript.

Reviewer #2

(Remarks to the Author)
The manuscript is comprehensive and appears complementary to the study published by the first author in “An experimental approach to disentangle neurodevelopmental and current factors of influence on brain and cognition: an intervention twin study” (Alzheimer's & Dementia, 2024).
I would be particularly interested in a clearer description of how the brainprint was constructed in the present work. More

illustrative details of this “generic” brainprint framework would be helpful, for example, a schematic or flowchart outlining the main steps, especially given that this represents a central component of the study.
While the manuscript is generally well structured and flows logically, the breadth of analyses makes it somewhat dense, at times giving the impression of two studies combined into one.
Additionally, review by a statistical expert would be beneficial given the methodological complexity.

Reviewer #3

(Remarks to the Author)
This paper investigates how environmental variation shapes cortical morphology using brainprint similarity — a composite index of cortical area, thickness, volume, and curvature — in monozygotic (MZ) and dizygotic (DZ) twin pairs. This work addresses an important question and has interesting findings. The design is elegant and the manuscript is well written. However, there are some concerns over the methodology and interpretation that should be addressed before publication.
1. Throughout the results, findings are reported with test statistics and p-values but without effect sizes (e.g., Cohen's d, partial eta-squared, or r). This is particularly concerning given the modest sample sizes involved — especially for the longitudinal VR training subset.
2. The BW-training time correlation (r = .286, p = .073) is described as 'a tendency.' For a p-value of .073, this framing is inappropriate.
3. Did the authors observe a difference in effect between the sexes? I would like to see the effect of sex in the model and possibly an interaction between sex and birthweight discordance.

Version 1:

Reviewer comments:

Reviewer #1

(Remarks to the Author)
Having re-read the revised manuscript, I can confirm that the authors have addressed all of my concerns. The results remain significant albeit attenuated even after adjusting for the intracranial volume. Their claims are supported, and I have no further issues to raise.

Reviewer #2

(Remarks to the Author)
From my perspective, the paper brings novelty and would make a valuable contribution to the field.
The authors have addressed my previous comments from the first stage of the review process, particularly by providing a clearer explanation of the brainprint concept and including a flowchart. I am therefore happy to recommend the current version of the manuscript for publication.

Reviewer #3

(Remarks to the Author)
The authors have adequately addressed my concerns, and I am satisfied with the revisions.

Open Access This Peer Review File is licensed under a Creative Commons Attribution 4.0 International License, which permits use, sharing, adaptation, distribution and reproduction in any medium or format, as long as you give appropriate credit to the original author(s) and the source, provide a link to the Creative Commons license, and indicate if changes were made.
In cases where reviewers are anonymous, credit should be given to 'Anonymous Referee' and the source.
The images or other third party material in this Peer Review File are included in the article's Creative Commons license, unless indicated otherwise in a credit line to the material. If material is not included in the article's Creative Commons license and your intended use is not permitted by statutory regulation or exceeds the permitted use, you will need to obtain permission directly from the copyright holder.
To view a copy of this license, visit https://creativecommons.org/licenses/by/4.0/

Responses to reviewer comments

We are very grateful for the helpful comments from all reviewers. We have revised the manuscript according to all comments and suggestions, conducting additional analyses and including additional information where requested (please see below and marked up changes in attached ms). We feel that all comments have helped improved the manuscript..

Reviewer #1 (Remarks to the Author):

In their manuscript, Walhovd et al. investigated a brain similarity index, brainprint, in a large sample of monozygotic (identical) and dizygotic (fraternal) twins at baseline and after exposure to intensive training in a virtual reality (VR)-based urban environment. They show that brainprints of monozygotic twins that differed in their birth weight, indicating differences in the prenatal environment, were less similar than those of their counterparts with more similar birth weights. Moreover, after the 10-week VR-intervention, they observed changes in brain curvature, showing training-induced plasticity, whereby monozygotic twins showed greater similarity after training, and the brainprints of dizygotic twins differed even more due to the intervention. This manuscript contributes to our understanding of how genetic influences and environmental factors interact to affect certain aspects of cortical brain features.

The study and analyses are extensive and well described. I would like to commend the authors for their innovative and demanding work, not least the ingenuity with which they named the VR-based city and designed its layout. Overall, this work and manuscript are of high quality. I only have a few minor comments that the authors may wish to consider.

Main comment

When calculating brainprints, are the area and volumetric features normalised for total intracranial volume? Given the distributions of the individual features (Figure 2D and Figure 4), it seems to me that the ICV may have a slight influence on the area, surface and curvature (as expected, not on the thickness). Even for the curvature, although relatively safe, nonlinear allometric scaling can still subtly alter the pattern of folding. Although MZ are safe, DZ might reflect larger variability.

Response:

We thank the reviewer for raising this point, which we agree is worth consideration. The primary brainprints capture overall morphological similarity, including global scaling effects that may themselves reflect early developmental influences. In the original analyses, area and volume features were not normalized for ICV before brainprint calculation. We think there are neurodevlopmental aspects closely related to ICV that we otherwise will miss. However, we completeøy agree that it is also importat to assess to whether the observed associations could be a simple function of intracranial scaling. Thus, while we have kept these

unnormalized brainprints as the primary phenotype, we have added an ICV-normalized sensitivity analysis. Specifically, in response to the reviewer's concern, we normalized area and volume features against SAMSEG-estimated total intracranial volume using region-specific allometric scaling exponents (i.e. allowing for nonlinear scaling). Median ICV-scaling exponents across regions were 0.656 for cortical area (2.5-97.5% range: 0.441-0.929) and 0.543 for cortical volume (0.223-0.875). Brainprint similarity was then recomputed using these ICV-normalized features and entered into the same birthweight-discordance model as in the original analyses. The birthweight-discordance x zygosity interaction was attenuated but remained significant after ICV normalization of area and volume features (t = 2.40, p = 0.018, partial r = 0.236). We further repeated the analysis including cortical curvature in the ICV normalization. As intuited by the reviewer, cortical curvature showed minimal ICV scaling (median exponent = 0.029), and the interaction again remained significant (t = 2.57, p = 0.012, partial r = 0.251). At the feature-category level, the MZ associations with birthweight discordance remained significant for area, volume, and curvature after ICV normalization, whereas thickness and the corresponding DZ associations remained non-significant (ref. Fig 2C). These analyses indicate that ICV-related scaling accounts for part, but not all, of the observed birthweight-discordance association.

We have added the following to the updated manuscript:

"Results, p.9

Because area and volume measures scale with intracranial volume, we repeated the global and category-level analyses after region-wise allometric ICV normalization of area and volume features, and in a second sensitivity analysis also normalized curvature. The BW discordance x zygosity interaction was attenuated but remained significant, and the MZ-specific category-level pattern was preserved (Table S3).

Supplementary materials, Table S3

Feature Handling	***ICV-scaling exponents***	***Full 272-feature brainprint interaction***	***Category-level pattern***
Raw original brainprints	Not normalized	t=4.01,<br><br>p=0.0001,<br><br>partial r=0.375	MZ: area t = -7.17, p < .0001, r = -0.66; volume t = -5.32, p < .0001, r = -0.55; curvature t = -3.196, p = 0.0021, r = -0.37; thickness n.s.<br><br>DZ: all categories n.s.
Area + volume ICV-normalized	Area beta = 0.656 [0.441, 0.929]<br><br>Volume beta = 0.543 [0.223, 0.875]	t=2.40,<br><br>p=0.018,<br><br>partial r=0.236	MZ: area t = -3.44, p = 0.001, r = -0.39; volume t = -2.72, p = 0.008, r = -0.32; curvature t = -3.2, p = 0.002, r = -0.37; thickness n.s.<br><br>DZ: all categories n.s.
Area + volume + curvature	Area and Volume as above;<br><br>Curvature beta = 0.029 [-0.121, 0.305]	t=2.57,<br><br>p=0.012,	MZ: area t = -3.44, p = 0.001, r = -0.39; volume t = -2.72, p = 0.008, r = -0.32; curvature t = -3.62, p = 0.0006, r = -0.41; thickness n.s.

ICV-normalized		partial r=0.251	DZ: all categories n.s.

Table S3. *ICV/allometric sensitivity analysis of the birthweight-discordance association with brainprint similarity. ICV-normalized features were adjusted using region-specific allometric exponents estimated as log(feature) ~ log(ICV) + (1 | twin_pair). Values in the exponent column are median beta [2.5-97.5% empirical range] across regions. Full-brainprint results show the birthweight-discordance ratio x zygosity interaction. Category-level results summarize the birthweight-discordance ratio association within zygosity. Full-brainprint models included zygosity, sex, smooth age, mean birthweight, birthweight-discordance ratio, and the birthweight-discordance ratio x zygosity interaction. Category-level models were fit separately within MZ and DZ pairs and included sex, smooth age, mean birthweight, and birthweight-discordance ratio. Full-brainprint N = 105 twin pairs; category-level MZ N = 71 pairs and DZ N = 34 pairs. n.s. indicates p > 0.05."*

Minor comments:

Abstract/Introduction
1. You introduce the term brainprint quite late; perhaps consider adding a general short explanation already in the abstract. (Up until the Methods section, I was wondering how it differs from or is connected to the BrainPrint.)

Response:

We agree and have revised the Abstract to define the term at first mention. This required minor reformatting of the abstract to stay under the word limit; the updated version now reads:

"How environmental variation shapes the human cerebral cortex remains incompletely understood. We compared cortical brainprints – individual profiles of regional cortical morphology – based on 210 twins (71 MZ and 34 DZ pairs, age 16-78 years) to distinguish prenatal, adult naturalistic and experimentally induced environmental variation from genetic contributions. Genetic effects were reflected in higher brainprint similarity within monozygotic (MZ) than dizygotic (DZ) pairs. Early environmental contributions were evident in lower brainprint similarity in MZ twin pairs with larger birthweight discordances, driven by area across the cortical ribbon. Later within-pair environmental differences in adult weight and lifestyle had minimal influence. Still, a 10-week virtual-reality navigation intervention revealed training-induced changes in the gray-white interface, with curvature and area changes supported by microstructural reconfigurations. In support of gene-environment interactions, relative brainprint similarity increased in MZ but diverged in DZ pairs following training. The results demonstrate that early-life environmental differences persistently shape cortical architecture even in genetically identical twins. Environmental influence in adulthood in the form of training can still affect similarity of twin brainprints at the grey-white-matter boundary. These findings show that distinct environmental influences at prenatal and adult stage are differentially expressed across cortical features within a genetically informed framework."

We have also clarified the distinction from Wachinger et al. in the Introduction (p. 4):

"A brainprint [38-40] (Fig. 1) was computed from 272 cortical features (68 each for area, thickness, volume, and curvature[41, 42]) and used as an index of structural brain similarity of MZ and DZ twins. Here, "brainprint" denotes a generic individual profile of cortical morphology, distinct from the specific BrainPrint/shapeDNA implementation of Wachinger and colleagues[40]."

(2.) p. 4, line 72: 'We examine how human cortical architecture – surface area, thickness, volume, and curvature holistically and feature-wise are related to different environmental factors and interrogate effects further using measures of cortical microstructure.' I find this sentence a little awkward. Perhaps consider rewriting it.

Response:

We agree and have revised the sentence for clarity. It now reads:

"We examine how human cortical architecture relates to environmental variation, both at the level of global morphology and across specific cortical features: surface area, thickness, volume, and curvature. We further use measures of cortical microstructure to interrogate effects observed in the macrostructural features."

(3.) p. 7, line 149 Perhaps consider rewriting this sentence: 'we included for analyses a series of lifestyle-related variables.'

Response:

We agree and have revised accordingly. The full sentence now reads:

"Beyond zygosity, we examined which within-pair differences in environmental contributions were most relevant for brainprint similarity by including birth weight and additional lifestyle-related variables."

Methods
(4.) What was the sample composition gender/sex-wise?

Response:

We apologize for omitting this information from the original Methods. We have now added the sex composition to the Sample section (p.25):

> *"The project was approved by the Regional Committee for Medical Research Ethics, South Norway, and all participants gave written informed consent. The full co-twin MRI sample reported here is a subsample of the full Set-to-change study sample and comprised 210 individuals from 105 same-sex twin pairs with MRI data for both twins (age range 16–78 years at baseline; 144 females, 66 males). The sample included 142 MZ twins from 71 pairs (98 females, 44 males) and 68 DZ twins from 34 pairs (46 females, 22 males)."*
>
> ---

And we have included a brief overview in the first sentence of the Results (p.5):

--

> *"We analyzed data from 71 MZ and 34 DZ same-sex twin pairs (age 16–78 years, mean age 37 years, 72 female pairs, 33 male pairs)."*

(5.) It would be interesting to see the associations with the cognitive/performance scores from the training, but I can imagine this is beyond the scope of this manuscript.

Response:

We definitely share the reviewer's interest in pursuing the performance scores from the training in greater detail, and this will be a focus for future work. We note that we already include a performance-linked analysis in the manuscript, by examining within-pair discordance in level reached during the VR task. This analysis showed that twins who differed

in task performance (maximum level reached) also showed lower post-training brainprint similarity. Table S2 now shows this with effect sizes:

Model 10 (Table S1): Training effects controlling for performance (level reached) discordances					
Full sample, 105 pairs	Brainprint similarity (longitudinal; full: 272 features)	Maxlevel difference x Training status 1	t = -2.39; partial r = -.154; η_p^2 = .024	< .0178	Brainprint turning less similar immediately post-train if one twin reaches different maximum level than the other.

Reviewer #2 (Remarks to the Author):

The manuscript is comprehensive and appears complementary to the study published by the first author in “An experimental approach to disentangle neurodevelopmental and current factors of influence on brain and cognition: an intervention twin study” (Alzheimer's & Dementia, 2024).
I would be particularly interested in a clearer description of how the brainprint was constructed in the present work. More illustrative details of this “generic” brainprint framework would be helpful, for example, a schematic or flowchart outlining the main steps, especially given that this represents a central component of the study.
While the manuscript is generally well structured and flows logically, the breadth of analyses makes it somewhat dense, at times giving the impression of two studies combined into one. Additionally, review by a statistical expert would be beneficial given the methodological complexity.

Response:

We thank the reviewer for highlighting this. The brainprint framework is indeed a central part of the study, and we appreciate suggestions for how to convey our approach more clearly to a wider audience. We have now added a new schematic overview as Supplementary Fig. 10, expanding the brainprint-construction step shown in Fig. 1D. The figure follows the pipeline from MRI input and cortical parcellation to feature-set construction, brainprint vectors, pairwise similarity calculation, and analysis output. To clarify the generality of the framework, the schematic distinguishes the full cortical macrostructural brainprint, feature-category brainprints, single-feature similarity analyses, and diffusion-based brainprints. We have also referenced this schematic in the Methods section where brainprint construction is described.

Methods, section "Brainprint analyses" (p.35) now includes:

“A schematic overview of the full, feature-category, single-feature, and diffusion brainprint workflows is provided in Fig. S10.”

Caption of Fig1D now includes the following sentence:

“See Fig. S10 for an expanded schematic of the brainprint construction framework across analysis levels.”

The new Figure S10, with caption, is shown below:

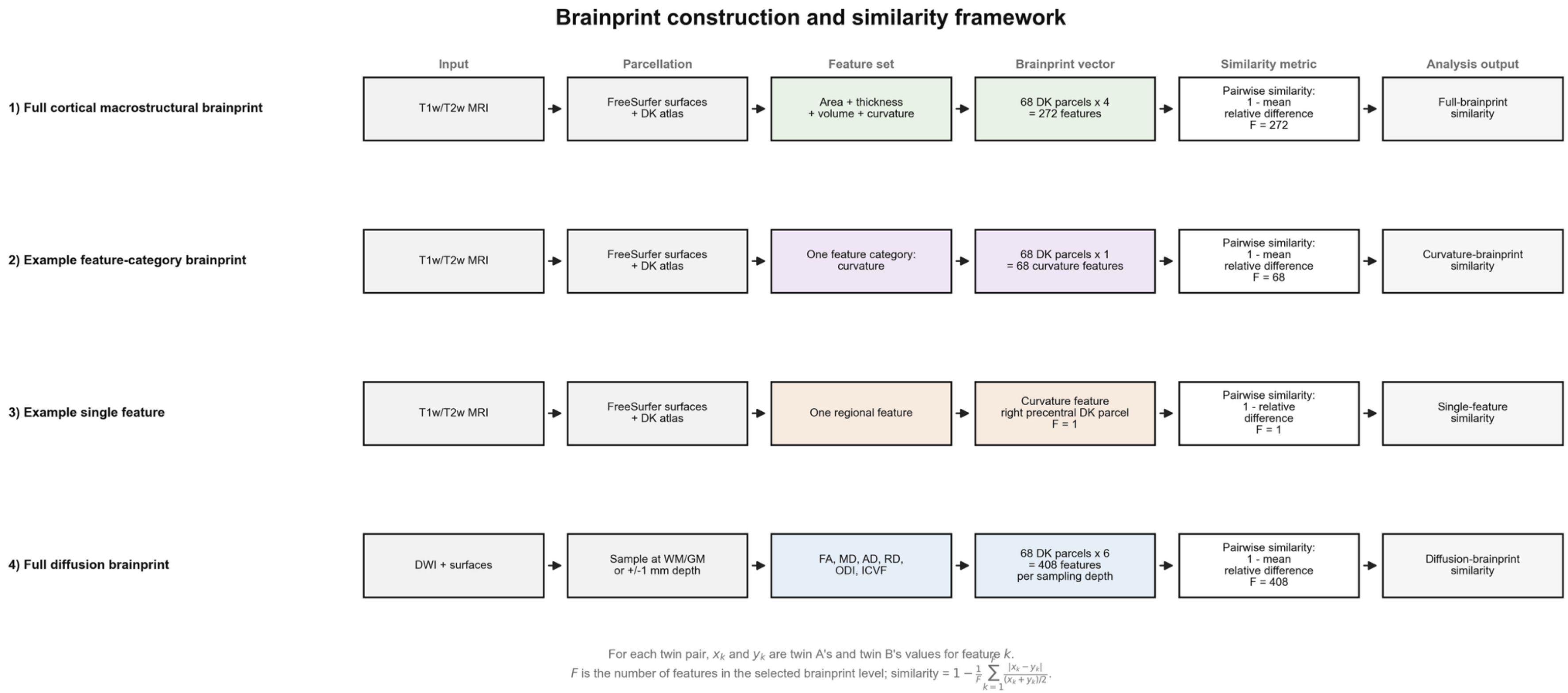

Fig. S10. Brainprint construction and similarity framework. Schematic overview of how brainprints were constructed at different levels of feature aggregation. For cortical macrostructure, regional features were extracted from FreeSurfer surfaces using the Desikan–Killiany atlas. The full cortical macrostructural brainprint comprised 272 features: 68 parcels for each of area, thickness, volume, and curvature. Feature-category brainprints used one feature class at a time, and single-feature analyses computed similarity for individual regional features. Diffusion brainprints were constructed from diffusion-derived metrics sampled relative to the gray–white matter boundary. For each twin pair, similarity was defined as one minus the mean relative difference across the selected feature set, where F denotes the number of features included in the corresponding brainprint. The schematic illustrates the common feature-construction and similarity framework; analysis-specific covariate adjustment and sensitivity analyses, including ICV/allometric normalization, are described in the Methods and applied as specified for each analysis.

We thank reviewer two for the suggestions, and hope this helpes further delinate the brainprint framework for readers.

Reviewer #3 (Remarks to the Author):

This paper investigates how environmental variation shapes cortical morphology using brainprint similarity — a composite index of cortical area, thickness, volume, and curvature — in monozygotic (MZ) and dizygotic (DZ) twin pairs. This work addresses an important question and has interesting findings. The design is elegant and the manuscript is well written. However, there are some concerns over the methodology and interpretation that should be addressed before publication.

1. Throughout the results, findings are reported with test statistics and p-values but without effect sizes (e.g., Cohen's d, partial eta-squared, or r). This is particularly concerning given the modest sample sizes involved — especially for the longitudinal VR training subset.

Response:

We agree with the reviewer that effect sizes are important. The reason we did not include effects sizes for allanyalsues in the manuscript, was to help readability as the analyses are many. We have now revised the Results and supplementary tables to report effect sizes more consistently. Several figures already displayed effect-size metrics, including Pearson/partial correlations for brainprint similarity and feature-wise partial-r maps/distributions. However, we agree that the numerical reporting in the Results and tables was incomplete. We have therefore expanded Table S2 so that the statistic column now reports partial r and partial η² for the key t-tested model terms. First rows shown here as example:

Sample (N)	***Dependent***	***Key term***	***Statistic***	***p***	***Notes / Figure***
**Model 1 (Table S1): Birth weight discordance and baseline brainprint similarity**					
Full sample; 105 pairs	Brainprint similarity (baseline; full: 272 features)	BW ratio x zygosity	$t = 4.01$; partial $r = .375$; $\eta_p^2 = .141$	$= .0001$	Core BW x zygo interaction; Fig. 2B/4A
Full sample; 105 pairs	Brainprint similarity (baseline; full: 272 features)	BW ratio (main effect)	$t = -5.33$; partial $r = -.474$; $\eta_p^2 = .225$	$< .0001$	BW effect (depends on zygo); Fig. 2B
Full sample; 105 pairs	Brainprint similarity (baseline; full: 272 features)	Zygosity (main effect)	$t = -5.31$; partial $r = -.473$; $\eta_p^2 = .224$	$< .0001$	Lower similarity in DZ; Fig 1A/2B

We also added effect sizes to the main text for the primary birthweight-discordance x zygosity effect and for the central longitudinal VR-training analyses, where – as correctly pointed out by the reviewer – sample size was most limited. Specifically, changes were done in:

Caption, Figure 2B:

"Brainprint similarity between twins at baseline decreased with increasing BW discordance in 71 MZ pairs, but not in 34 DZ pairs (Fig. 2B). The primary test was a generalized additive model including BW discordance, zygosity, and their interaction (adjusting for age (smooth), sex, and mean BW; Table S1: Model 1), which showed a significant BW discordance × zygosity interaction ($t = 4.01$, $p = .0001$, partial $r = .375$; Table S2: Model 1)."

--

Results, section "Cortical changes in response to navigation training in virtual reality", p.13:

"To provide a strict test of training-related change, we first restricted analyses to twin pairs with valid training engagement and complete pre- and post-training MRI (30 MZ and 14 DZ pairs). Using the full brainprint, we observed a significant training x zygosity interaction immediately post-training ($t = -2.324$, $p = .022$, partial $r = -.206$), indicating that MZ twins became more similar relative to DZ twins after training (Table S2: Model 5). This effect was attenuated at follow-up ($t = -1.287$, $p = 0.20$, partial $r = -.116$)."

Results, section "Cortical changes in response to navigation training in virtual reality", p.16:

"We investigated the generalizability of training-related effects detected in the curvature brainprints across the cortical mantle (30 MZ and 14 DZ pairs; no non-training). The training status x zygosity interaction in curvature similarity was present

for measures defined at the white matter surface (t = -3.312, p = .0012, partial r = -.287), whereas no corresponding effect was observed for pial-surface curvature similarity (t = 1.313, p = .1915, partial r = .118).”

We have also added this sentence towards the end of the Methods, section "Statistical analyses" (p. 38):

“For model terms tested with t-statistics, we report partial r and partial η^2 as effect-size measures, derived from the model t-value and residual degrees of freedom. “

---

2. The BW-training time correlation (r = .286, p = .073) is described as 'a tendency.' For a p-value of .073, this framing is inappropriate.

Response:

We agree and have removed the "tendency" wording. The sentence now states (p. 16)

“Moreover, we observed evidence consistent with developmental contributions to training performance: in 41 MZ twin pairs with valid training data (independent of post-training MRI status), birthweight ratio (heaviest/lightest co-twin) correlated positively with difference in level reached (Pearson r = .347, p = .028), whereas the corresponding association with within-pair difference in training time did not reach statistical significance (r= .286, p=.073).”

---

3. Did the authors observe a difference in effect between the sexes? I would like to see the

effect of sex in the model and possibly an interaction between sex and birthweight discordance.

Response:

We thank the reviewer for the comment. While we agree that sex differences could be of interest, as these are same-sex twin pairs, there are some restrictions to what can meaningfully be anlyzed, and we want tp be cautious in this regard. We apologize for not making information on sex sufficently clear from the original Methods. We have now added the sex composition to the Sample section (p. 25):

> *"The full co-twin MRI sample reported here is a subsample of the full Set-to-change study sample and comprised 210 individuals from 105 same-sex twin pairs with MRI data for both twins (age range 16–78 years at baseline; 144 females, 66 males). The sample included 142 MZ twins from 71 pairs (98 females, 44 males) and 68 DZ twins from 34 pairs (46 females, 22 males)."*
>
> *----*

And we have added a brief overview at the start of the Results (p.5):

> *---*
>
> *"We analyzed data from 71 MZ and 34 DZ same-sex twin pairs (age 16–78 years, mean age 37 years, 72 female pairs, 33 male pairs)."*

While there are limitations, we have now upon reveower request, also tested whether sex influenced the birthweight-discordance association. Because all included twin pairs were same-sex pairs, sex becomes a pair-level covariate in these analyses. In the original birthweight-discordance model, main effect of sex was not significant (male vs female: $t = -0.61$, $p = 0.54$, partial $r = -0.06$). We then added a sex x birthweight-discordance interaction to test whether the association differed between female-female and male-male pairs; this interaction was also not significant ($t = -0.59$, $p = 0.56$, partial $r = -0.06$). Finally, although the subgroup sizes make a formal three-way test underpowered, we also tested the sex x zygosity x birthweight-discordance interaction; this was likewise not significant ($t = -0.80$, $p = 0.42$, partial $r = -0.08$). Thus, we found no evidence that the birthweight-discordance association differed by sex. We have added to the Results paragraph describing the effects of birthweight discordance on brainprint similarity (p. 9):

"We first evaluated how baseline brainprint similarity was associated with prenatal environmental variance, indexed by birth weight (BW) discordance across all 105 twin pairs. Brainprint similarity decreased with increasing BW discordance, but this effect depended on zygosity: greater BW discordance was associated with lower brainprint similarity in MZ twins, whereas no corresponding association was observed in DZ twins (Fig. 2B; Table S2: Model 1). Sex was not significantly associated with brainprint similarity in this model ($p = 0.54$), and adding a sex x BW-discordance interaction provided no evidence that the BW-discordance association differed between female and male twin pairs ($p = 0.56$)."
