# Supplementary material for: Feature-specific environmental contributions to human cortical architecture revealed by twin brainprints
Source: Commun Biol. 2026 Jul 20;9:1503. doi: 10.1038/s42003-026-10707-w (PMC13752308; doi:10.1038/s42003-026-10707-w)
Supplement: Supplementary file 2 [file 42003_2026_10707_MOESM2_ESM.pdf]

Supplementary Material

for

Feature-specific environmental contributions to human cortical architecture revealed by twin brainprints

Kristine B Walhovd*, Anne Cecilie Sjøli Bråthen, Knut Overbye, Jonas Kransberg, Øystein Sørensen, Pablo F. Garrido, Inge K. Amlien, I., Jose-Luis Alatorre-Warren, Athanasia M. Mowinckel, Maksim Slivka, Nikolai O. Czajkowski, Yunpeng Wang, Paulina Due-Tønnessen, Jennifer R. Harris, Martin Lövdén, Didac Vidal-Pineiro, Lars Nyberg, Anders M. Fjell, Markus H. Sneve.

***Corresponding Author:** Kristine B. Walhovd, Center for Lifespan Changes in Brain and Cognition, Department of Psychology, University of Oslo, POB 1094 Blindern, 0317 Oslo, Norway, k.b.walhovd@psykologi.uio.no, +47 46824662

This file includes:

Supporting text

Tables S1 to S3

Figures S1 to S10

Supporting results

GA(M)M	Formula	Random Effects
1	Brainprint similarity ~ zygosity + sex + s(age) + mean birth weight + birth weight discordance + zygosity * birth weight discordance	NA (one observation per twin pair)
2	Global cortical area ~ birth weight + sex + s(age at baseline)	~(1 | twin pair)
3	Global cortical area ~ birth weight + sex + s(age at baseline) + icv	~(1 | twin pair)
4	Global cortical area ~ birth weight + sex + s(age at baseline) + icv + BMI	~(1 | twin pair)
5	Brainprint similarity ~ training status + zygosity + s(age) + zygosity * training status	~(1 | twin pair)
6	Global cortical curvature~ training status + sex + s(age at baseline) + time + age at baseline : time	~(1 | id)
7	Global cortical area~ training status + sex + s(age at baseline) + time + age at baseline : time	~(1 | id)
8	Brainprint similarity ~ training status + zygosity + s(age) + zygosity * training status + diffusion similarity + diffusion similarity * training status	~(1 | twin pair)
9	Brainprint similarity ~ training status + zygosity + training time difference + s(age) + training status * training time difference + zygosity * training status	~(1 | twin pair)
10	Brainprint similarity ~ training status + zygosity + max level difference + s(age) + training status * max level difference + zygosity * training status	~(1 | twin pair)

Table S1. Generalized additive (mixed) models (GA(M)M) formulas run. Main analyses are shown in shaded rows, follow-up analyses in white.

Explanation of terms: zygosity: ordered categorical, 1 = MZ, 2 = DZ; sex: male/female; “s(age)”: smooth-term of twin1 age in years (with 5 decimal points) at measurement; “age at

baseline": age in years (with 5 decimal points) at first measurement; "time": time since baseline, i.e. age in years minus minimum age at measurement; "birth weight": birth weight of single individual in grams, "mean birthweight": (twin 1 BW + twin 2 BW)/2; "birth weight discordance": If ratio, BW heaviest twin / BW lightest twin. If difference, BW heaviest twin – BW lightest twin; "BMI": current body mass index; "training status": ordered categorical, 0 = pre-training, 1 = immediately post-training, 2 = delayed post-training. "training time difference": z-normalized absolute difference in training times between twins in a pair.

Sample (N)	*Dependent*	*Key term*	*Statistic*	*p*	*Notes / Figure*

Model 1 (Table S1): Birth weight discordance and baseline brainprint similarity

Full sample; 105 pairs	Brainprint similarity (baseline; full: 272 features)	BW ratio x zygosity	t = 4.01; partial r = .375; η_p^2 = .141	= .0001	Core BW x zygo interaction; Fig. 2B/4A
Full sample; 105 pairs	Brainprint similarity (baseline; full: 272 features)	BW ratio (main effect)	t = -5.33; partial r = -.474; η_p^2 = .225	< .0001	BW effect (depends on zygo); Fig. 2B
Full sample; 105 pairs	Brainprint similarity (baseline; full: 272 features)	Zygosity (main effect)	t = -5.31; partial r = -.473; η_p^2 = .224	< .0001	Lower similarity in DZ; Fig 1A/2B
MZ sample; 71 pairs	Brainprint similarity (baseline; area only: 68 features)	BW ratio (main effect)	t = -7.17; partial r = -.662; η_p^2 = .438	< .0001	↑MZ BW discordance ↓Brainprint similarity; Area; Fig. 2C
MZ sample; 71 pairs	Brainprint similarity (baseline; volume only: 68 features)	BW ratio (main effect)	t = -5.32; partial r = -.548; η_p^2 = .300	< .0001	↑MZ BW discordance ↓Brainprint similarity; Volume; Fig. 2C
MZ sample; 71 pairs	Brainprint similarity (baseline; curvature only: 68 features)	BW ratio (main effect)	t = -3.2; partial r = -.366; η_p^2 = .134	= .002	↑MZ BW discordance ↓Brainprint similarity; Curvature; Fig. 2C

Model 2 (Table S1): Birth weight and global cortical area (WM vs pial; full and MZ samples)

Full sample; 210 individuals	Global cortical area (baseline; quantified at WM surface)	BW (main effect)	t = 4.12; partial r = .276; η_p^2 = .076	< .0001	↑BW ↑WM Surface Area
MZ sample; 142 individuals	Global cortical area (baseline; quantified at WM surface)	BW (main effect)	t = 7.89; partial r = .558; η_p^2 = .311	< .0001	↑BW ↑WM Surface Area; Genetic control
Full sample; 210 individuals	Global cortical area (baseline; quantified at PIAL surface)	BW (main effect)	t = 4.21; partial r = .281; η_p^2 = .079	< .0001	↑BW ↑Pial Surface Area
MZ sample; 142 individuals	Global cortical area (baseline; quantified at PIAL surface)	BW (main effect)	t = 8.10; partial r = .568; η_p^2 = .322	< .0001	↑BW ↑Pial Surface Area; Genetic control

Model 3 (Table S1): Birth weight and global cortical area controlling for intracranial volume (ICV)

Full sample, 210 individuals	Global cortical area (baseline; quantified at WM surface)	BW (main effect)	t = 2.23; partial r = .154; η_p^2 = .024	= .0268	↑BW ↑WM Surface Area, controlling for ICV
MZ sample; 142 individuals	Global cortical area (baseline; quantified at WM surface)	BW (main effect)	t = 4.78; partial r = .378; η_p^2 = .143	< .0001	↑BW ↑WM Surface Area, controlling for ICV; Genetic control

Model 4 (Table S1): Birth weight and global cortical area controlling for ICV and BMI

Full sample, 210 individuals	Global cortical area (baseline; quantified at WM surface)	BW (main effect)	t = 2.00; partial r = .139; η_p^2 = .019	= .0467	↑BW ↑WM Surface Area, controlling for ICV and BMI
MZ sample; 142 individuals	Global cortical area (baseline; quantified at WM surface)	BW (main effect)	t = 4.39; partial r = .352; η_p^2 = .124	< .0001	↑BW ↑WM Surface Area, controlling for ICV and BMI; Genetic control

Sample (N)	***Dependent***	***Key term***	***Statistic***	***p***	***Notes / Figure***
		**Model 5 (Table S1): Training status × zygosity effects on longitudinal brainprint similarity**			
Trainers-only; 44 pairs	Brainprint similarity (longitudinal; full: 272 features)	Training status 1 x zygosity	t = -2.32; partial r = -.206; $\eta_p^2 = .042$	= .022	Strictest training test: MZ more similar relative to DZ immediately following training, Fig 3B
Trainers-only; 44 pairs	Brainprint similarity (longitudinal; full: 272 features)	Training status 2 x zygosity	t = -1.29; partial r = -.116; $\eta_p^2 = .013$	= .2005	Strictest training test: Attenuation at follow-up, Fig 3B
Trainers-only; 44 pairs	Brainprint similarity (longitudinal; full: 272 features)	Status 1 (main effect)	t = 0.89; partial r = .081; $\eta_p^2 = .007$	= .372	No immediate main effect of training (independent of zygo)
Trainers-only; 44 pairs	Brainprint similarity (longitudinal; full: 272 features)	Status 2 (main effect)	t = 0.11; partial r = .010; $\eta_p^2 < .001$	= .913	No postponed main effect of training (independent of zygo)
Full sample, 105 pairs	Brainprint similarity (longitudinal; full: 272 features)	Training status 1 x zygosity	t = -2.78; partial r = -.178; $\eta_p^2 = .032$	= .006	Full-sample replication: MZ more similar relative to DZ immediately following training
Full sample, 105 pairs	Brainprint similarity (longitudinal; full: 272 features)	Training status 2 x zygosity	t = -1.51; partial r = -.098; $\eta_p^2 = .010$	= .131	Full-sample replication: Attenuation at follow-up
Trainers-only; 44 pairs	Brainprint similarity (longitudinal; curvature only: 68 features, quantified at WM surface)	Training status 1 x zygosity	t = -3.31; partial r = -.287; $\eta_p^2 = .082$	= .0012	Location-of-training effect; present at WM surface
Trainers-only; 44 pairs	Brainprint similarity (longitudinal; curvature only: 68 features, quantified at PIAL surface)	Training status 1 x zygosity	t = 1.31; partial r = .118; $\eta_p^2 = .014$	= .1915	Location-of-training effect; absent at PIAL surface
Full sample, 105 pairs	Brainprint similarity (longitudinal; curvature only: 68 features, quantified at WM surface)	Training status 1 x zygosity	t = -4.37; partial r = -.273; $\eta_p^2 = .075$	< .0001	Full-sample replication: Location-of-training effect; present at WM surface
Full sample, 105 pairs	Brainprint similarity (longitudinal; curvature only: 68 features, quantified at PIAL surface)	Training status 1 x zygosity	t = 1.27; partial r = .082; $\eta_p^2 = .007$	= .2051	Full-sample replication: Location-of-training effect; absent at PIAL surface
		**Model 6 (Table S1): Global cortical curvature change as a function of training status**			
Trainers-only; 86 individuals	Global cortical curvature (longitudinal; quantified at WM surface)	Training status 1 (main effect)	t = 1.07; partial r = .068; $\eta_p^2 = .005$	= .2830	
Trainers-only; 86 individuals	Global cortical curvature (longitudinal; quantified at PIAL surface)	Training status 1 (main effect)	t = -0.35; partial r = -.023; $\eta_p^2 = .001$	= .724	
Full sample; 210 individuals	Global cortical curvature (longitudinal; quantified at WM surface)	Training status 1 (main effect)	t = 2.10; partial r = .096; $\eta_p^2 = .009$	= .0360	
Full sample; 210 individuals	Global cortical curvature (longitudinal; quantified at PIAL surface)	Training status 1 (main effect)	t = 0.71; partial r = .032; $\eta_p^2 = .001$	= .481	

Sample (N)	*Dependent*	*Key term*	*Statistic*	*p*	*Notes / Figure*
**Model 7 (Table S1): Global cortical area change as a function of training status**					
Trainers-only; 86 individuals	Global cortical area (longitudinal; quantified at WM surface)	Training status 1 (main effect)	t = 1.09; partial r = .069; η_p^2 = .005	= .279	
Trainers-only; 86 individuals	Global cortical area (longitudinal; quantified at PIAL surface)	Training status 1 (main effect)	t = -1.46; partial r = -.092; η_p^2 = .009	= .146	
Full sample; 210 individuals	Global cortical area (longitudinal; quantified at WM surface)	Training status 1 (main effect)	t = 2.62; partial r = .119; η_p^2 = .014	= .0090	
Full sample; 210 individuals	Global cortical area (longitudinal; quantified at PIAL surface)	Training status 1 (main effect)	t = -0.13; partial r = -.006; η_p^2 < .001	= .900	
**Model comparison: Model 8 vs Model 5 (Table S1): model improvement from microstructural information**					
DWI sample; 206 individuals	Curvature similarity (longitudinal; quantified at WM surface)	Diffusion sampling depth = 1mm into WM	χ^2 = 12.90	= .0277 (FDR)	ΔadjR²=6.3%; ΔAIC=6.9; Fig. 3E
DWI sample; 206 individuals	Curvature similarity (longitudinal; quantified at WM surface)	Diffusion sampling depth = at WM/GM	χ^2 = 18.66	=.0035 (FDR)	ΔadjR²=7.7%; ΔAIC=12.7; Fig. 3E
DWI sample; 206 individuals	Curvature similarity (longitudinal; quantified at WM surface)	Diffusion sampling depth = 1mm into GM	χ^2 = 8.24	= .152 (FDR)	ΔadjR²=3.2%; ΔAIC=2.2; Fig. 3E
**Model 9 (Table S1): Training effects controlling for training time discordances**					
Full sample, 105 pairs	Brainprint similarity (longitudinal; full: 272 features)	Training time difference x Training status 1	t = -7.20; partial r = -.424; η_p^2 = .180	< .0001	Brainprint turning less similar immediately post-train if one twin trains more than the other.
**Model 10 (Table S1): Training effects controlling for performance (level reached) discordances**					
Full sample, 105 pairs	Brainprint similarity (longitudinal; full: 272 features)	Maxlevel difference x Training status 1	t = -2.39; partial r = -.154; η_p^2 = .024	< .0178	Brainprint turning less similar immediately post-train if one twin reaches different maximum level than the other.

Table S2. Key outputs from GA(M)M analyses. For each model specified in Table S1, the table lists the sample (pairs or individuals), dependent variable, key term(s) referenced in the main text/figures, corresponding test, effect sizes, and p-value. Shaded rows indicate tests involving the full twin pair sample of 105 pairs.

Feature Handling	*ICV-scaling exponents*	*Full 272-feature brainprint interaction*	*Category-level pattern*
Raw original brainprints	Not normalized	t=4.01,<br><br>p=0.0001;<br><br>partial r=0.375	MZ: area t = -7.17, p < .0001, r = -0.66; volume t = -5.32, p < .0001, r = -0.55; curvature t = -3.196, p = 0.0021, r = -0.37; thickness n.s.<br><br>DZ: all categories n.s.
Area + volume ICV-normalized	Area beta = 0.656 [0.441, 0.929]<br><br>Volume beta = 0.543 [0.223, 0.875]	t=2.40,<br><br>p=0.018,<br><br>partial r=0.236	MZ: area t = -3.44, p = 0.001, r = -0.39; volume t = -2.72, p = 0.008, r = -0.32; curvature t = -3.2, p = 0.002, r = -0.37; thickness n.s.<br><br>DZ: all categories n.s.
Area + volume + curvature ICV-normalized	Area and Volume as above;<br><br>Curvature beta = 0.029 [-0.121, 0.305]	t=2.57,<br><br>p=0.012,<br><br>partial r=0.251	MZ: area t = -3.44, p = 0.001, r = -0.39; volume t = -2.72, p = 0.008, r = -0.32; curvature t = -3.62, p = 0.0006, r = -0.41; thickness n.s.<br><br>DZ: all categories n.s.

Table S3. ICV/allometric sensitivity analysis of the birthweight-discordance association with brainprint similarity. ICV-normalized features were adjusted using region-specific allometric exponents estimated as log(feature) ~ log(ICV) + (1 | twin_pair). Values in the exponent column are median beta [2.5-97.5% empirical range] across regions. Full-brainprint results show the birthweight-discordance ratio x zygosity interaction. Category-level results summarize the birthweight-discordance ratio association within zygosity. Full-brainprint models included zygosity, sex, smooth age, mean birthweight, birthweight-discordance ratio, and the birthweight-discordance ratio x zygosity interaction. Category-level models were fit separately within MZ and DZ pairs and included sex, smooth age, mean birthweight, and birthweight-discordance ratio. Full-brainprint N = 105 twin pairs; category-level MZ N = 71 pairs and DZ N = 34 pairs. n.s. indicates p > 0.05.

Supporting methods

Study design and sample

Genotyping: Briefly, the DNA samples were genotyped by the Global Screening Array (Illumina, CA). The genotypes were quality checked and imputed to the 1000 Genomes EUR population using our previous established protocol[1]After further quality check of imputed genotypes, the PLINK2 software[2] was used to compute the kinship coefficients based on autosomal variants which have a minor allele frequency >0.1 and are not correlated with each other ($r < 0.1$). Monozygotic twins were defined as those whose kinship coefficients > 0.4 and dizygotic twins are > 0.2 and < 0.3.

Data collection: The data collection for this study started in 2019 and ran throughout 2023, the prolonged period being due to corona restrictions during part of the project. The reasons for dropping out of training in the VR lab, MRI scanning, or project participation overall, thus may represent a mix of direct physical hindrance, and individual motivation and anxiety, the relative portions of which we unfortunately cannot meaningfully calculate, as differing restrictions and advices applied per participant timing, address, age group and health status. Hypotheses for an fMRI paradigm for the study as well as select structural ROIs (including hippocampus, entorhinal cortex) were preregistered in 2020 on https://aspredicted.org, but the methods used in the current paper were not pre-registered, as prolonged corona restrictions in part led to a much prolonged sample recruitment period, a sample of half the desired and registered size, and somewhat altered methods overall. Given this, along with methodological development in image processing, it was decided to use multi-feature cortical brainprints for the present study. We did not have hypotheses regarding brainprints in the initial preregistration, as we started using this methodology only later.

Cybercycle training program

The Cybercycle training program consisted of two variants designed to engage participants in navigating a virtual city called *Plasti City*: A Virtual Reality (VR) version conducted in our VR lab and an online/laptop-based version for playing remotely. These variants share similar tasks with slight differences in control schemes, and participants were asked to engage in both, ideally coming to the VR lab 2-3 times a week, and practicing the task at home 2-3 additional days a week. During the initial VR lab sessions, participants focused on adapting to the immersive VR environment. These sessions were deliberately kept brief at first and began with simple cycling along a virtual road, without any additional tasks (see below for details). Participants were encouraged to practice frequently but were advised to keep immersive VR sessions shorter (e.g., 20 minutes and no more than an hour) than the online or laptop-based sessions to reduce the risk of cyber-sickness (i.e., motion sickness). A series of tasks were presented to the participants, and unless otherwise noted, tasks were the same both in the VR lab and online/at laptops. Reporting on the results of these additional tasks is beyond the scope of the present paper, but for a full depiction of the intervention, they are also described below.

Description of the virtual city Plasti City: The virtual city used in the training program, Plasti City, is not based on any existing city and was manually built within Unreal Engine. The city is made up of several larger areas that are visually distinct, including a modern downtown in the south, a less dense suburb in the east, and a futuristic metropolis in the north. The total size of the city is roughly $5km^2$ (Best estimate: $4.96km^2$). The north and south side of the city are split by a canal district. A coast follows the southern and western edges of the city.

Equipment: For immersive VR sessions, participants were seated on a standard mountain bike which was affixed to a FLUID^2 Indoor Bike Trainer. The FLUID^2 Trainer is a progressive resistance roller that allows for a realistic biking experience. For safety and stability during the experiment, participants were also equipped with a Woodway chest-shoulder harness. This harness, typically utilized for heart rate monitoring and safety during treadmill exercises, was modified for our purposes. It was securely attached to an overhead anchor point, allowing participants to maintain balance on the stationary bike without risk of falling. All virtual navigation tasks were conducted using two types of head-mounted display (HMD) systems: the HTC Vive Pro and the HTC Vive Cosmos Elite. The HTC Vive Pro HMD offers a high-resolution display (2880 x 1600 pixels) with a 110-degree field of view and a refresh rate of 90 Hz, whereas the HTC Vive Cosmos Elite features a combined pixel resolution of 2880 x 1700. Both HMDs provide a fully immersive 3D experience. Positional tracking was achieved through the SteamVR Tracking system, which ensures precision within the virtual space by using two base stations positioned in the testing area. A Vive controller attached to the bike's handlebar and a Vive Tracker strapped inside the rear wheel of the bike allowed for tracking steering and wheel rotation respectively. All equipment was interfaced with a computer system equipped with an NVIDIA GeForce GTX 1080 Ti graphics card to render the virtual environment in real-time without perceptible latency. The virtual environment for the navigation tasks was developed using Unreal Engine 4.22.

Tasks in Plasti City

<u>Main navigation task:</u> In this task, participants are presented with a 3D model of a unique city building or feature. The model appears within a translucent globe, floating above the handlebar. It slowly rotates to allow participants to view it from all angles. The model remains visible until the participant reaches and successfully identifies the displayed target.

To confirm target identification, participants must approach the target closely and press a button on the handlebar controller. The required distance is similar for all targets but may vary slightly. We have manually placed the "target area" to encompass the surrounding roads effectively (mean = 4.5 m, range: 3.5–6.9 m), see Figure S7. When a participant correctly identifies a target, their score is displayed on the screen. The scoring per trial, which is used for "leveling-up" in the game, represents the participant's travel distance from the edge of the previous target's area (or starting location for the first target) to the edge of the current target's area, divided by the optimal distance between these points. The optimal distance is calculated using Unreal Engine's native Navigation Mesh System. Scores of 1.3 or less (less than 30% over optimal distance) mark a successful trial, displayed in green with a consonant sound. Unsuccessful trials show the text in red with a dissonant sound. After identifying a target, the target model above the handlebar is replaced with the next target model.

Road Navigation: Participants can only navigate the city roads. If a participant veers off the road, the bike is instantly teleported back to its position and orientation from four meters ago. The bike's speed before teleportation is maintained to minimize discomfort caused by simulated acceleration in VR.

Retrospective memory task: Starting in the fifth week of training for each group, the retrospective memory task is enabled. Participants receive a text description of the task, followed by a sequence of three navigation targets displayed alongside images of objects, for 5 seconds each. Participants must remember which targets were among the three and which specific images were shown. When they encounter one of the three targets during regular navigation, they must press a separate button from the main task. Incorrect button presses result in an error message, but there is no opportunity for correction. Mistakes still count as

hits in the main navigation task. Correct button presses lead to a 3x3 grid of possible objects, one of which matches the initial image. In VR, participants select the target by turning their head toward the object, while the laptop version involves clicking with a mouse. Participants receive immediate feedback on the correctness of their answer. Six categories of images are used: Tools, fruit, hats, animals, furniture, and vehicles, each containing six subcategories, resulting in 216 possible target pictures. Picture selection depends on task difficulty, with eight levels: Difficulty Levels: 1. Six categories, 2. Three categories, 3. Two categories, 4. One category, 5. Three subcategories, 6. Two subcategories, 7. One subcategory, 8. One subcategory (grayscale). For example, at difficulty level 1, six pictures are selected from different categories. Level 2 involves two pictures from each of three categories. Level 4 uses pictures from the same category but different subcategories. Level 7 selects pictures from the same subcategory, while level 8 maintains the subcategory but presents images in grayscale. Task difficulty increases as participants perform well, ensuring challenges adapt to their progress. If participants ever had a 50% or higher accuracy on the previous six retrospective memory task presentations (counted across sessions), the difficulty level would increase by one for the next session. Starting difficulty was changed to level 4 based on preliminary data, indicating the task was too easy.

<u>Passive Prospective Memory Task:</u> Starting in the seventh week of training, the passive prospective memory task is enabled. In this task, many stationary 3D cars are introduced into the virtual city environment. The layout of these cars is unique for each session, generated using a randomly seeded procedural algorithm. The algorithm places the cars at semi-regular intervals on both sides of the road. We utilize different visual models for these cars, ensuring they are visually distinct from one another. After participants begin the experiment and receive instructions, they are shown one randomly selected car model that they must watch

out for during regular navigation. This car model is displayed, slowly rotating in front of them for 10 seconds. Participants are instructed that if they encounter one of these target cars while navigating the city, they should press the designated target button. When pressing the target button while next to a car the participant gets shown a text prompt indicating whether they selected the correct car or not. If the participant passes by a target car without pressing a button, they receive a text prompt telling them they missed it.

<u>Active Prospective Memory Task (Time-Based):</u> In the ninth week of training, the active prospective memory task becomes active. This task is exclusively available during virtual reality (VR) sessions and is not included in the at-home laptop-based sessions. In this task, participants are instructed to press a specific button on their VR controller when a certain amount of time has passed during a training session. The target time is randomized, ranging from a whole number between 3 and 9 minutes. This target time is displayed on a virtual screen within the VR environment each time a session begins. The current time in minutes and seconds is constantly displayed on the handlebar of the virtual bike. Starting in the tenth week, there is a modification to this task. The time display is no longer constantly visible but requires participants to press a separate button on the controller for it to appear. Participants are instructed to minimize the number of times they press this button, as each press results in a score reduction equivalent to a ten-second time penalty.

Difficulty Levels and "Leveling Up"

Both the main navigation task and the retrospective memory tasks were designed with adaptive difficulty. This means that the tasks become more challenging as participants perform well. In the main navigation task, increased difficulty entails expanding the navigable area and increasing the number of potential targets within the city. This is achieved by

removing virtual barriers that initially block off certain streets. The city is divided into 13 areas, with the first unlocked at the start. Additional areas are unlocked based on participant performance. To trigger an increase in difficulty, a participant must reach three targets consecutively with a deviance from the path below the success criteria, as described below. Only targets from the latest unlocked area of the city are considered in this evaluation, ensuring that participants are sufficiently familiar with the current city area before more areas become accessible. To ensure that participants encounter new targets and have the chance to "level up" in any given session, at least three out of the first ten targets are always from the latest unlocked city level. In the retrospective memory task, difficulty (or "level") is increased if participants correctly select three out of the latest five items across multiple sessions.

Behavioral data from Plasti City

Behavioral data from the tasks were collected online and directly from a set of identical laptops that participants borrowed and kept at home during the training period. Technical errors lead to a small portion of data known to be lost. Data from the task was saved in two ways: a long “continous log” of participants’ movement and input during the task and a shorted “event log”, only logging specific events such as button presses. There was a minor number of sessions for which the continous logs were saved, but not event logs enabling reconstruction of navigation and target count from the continous logs, and a few instances were observed where files lacked between levels, but the problem source could not be recreated. Participants in the project were initially able to log in and play online for their homework. However, due to experiences of some technical difficulties with online data collection for the homework tasks, it was after an initial period in the project decided that all participants should get identical laptops at which all data were automatically saved for collection post-training period. For the entire project period, 1189 online, 2304 laptop, and

1734 VR training sessions were recorded. Participants did themselves report on level reached in homework for correct placement of level each time they came to train in the VR lab. Homework data was retrieved from the laptops post-training. The levels saved from homework and the levels saved as trained in the VR-lab corresponded well (correlation of r =.94), and as a modest amount of data were lost due to technical difficulties, the highest level recorded played, whether that be at home or in the VR-lab, was used for analyses of “level reached”. However, for one set of DZ twins, there was no homework training data. These twins reported levels 4 and 3, respectively, and hence navigated accordingly at levels in VR. The pair was included as trainers in analyses, since they were recorded to have trained equal to the mean of the sample in immersive VR (4.27 and 5.39 hours of goal-directed navigation, targets counts: 242 and 226, mean relative deviance from shortest paths: 0.21 and 0.45, respectively). For all other training participants, both homework and immersive VR-data were retrieved and used in analyses of training times and level reached.

The measure of deviance from shortest path between two consecutive targets is estimated after transforming the city map into a skeletonized image, where all the streets are reduced to a 1-pixel-width representation. The shortest path is then determined using the Minimum Cost Path (MCP) approach, with the distance measured in pixels. The current participant’s path is also transformed into this same space, matching every position in the city to the closest skeletonized pixel. The deviance from shortest path is calculated by the difference between the participant’s path and the shortest path. The relative deviance from shortest path refers to the ratio between the deviance and the length of the shortest path. Both the skeleton transformation and the MCP algorithm are implemented using Python’s scikit-image package (https://doi.org/10.7717/peerj.453).

Precision and Sensitivity of brainprint similarity.

We leveraged data from N = 30 twin pairs (8 DZ), each with two passive scans (i.e. scans with no training in between), to assess the reliability of brainprint similarity. Intraclass correlation analysis indicated excellent reliability, with ICC(2,1) = 0.947 (CI: 0.891 – 0.974) (Figure S9A). The reliability was comparable when the analyses was restricted to MZ twins (N = 22 pairs) (ICC(2,1) = 0.929 (CI: 0.845 – 0.969)) (psych r-package). The within subject-measurement error – defined as the random variability in brainprint similarity across repeated scans – was also notably low (Sw= 0.0028) (Figure S9B). Given that brainprint similarity is a composite measure, these values reflect a remarkable level of precision. Additionally, we conducted a sensitivity analysis to determine the range of effect sizes that could be reliably detected given our sample size, corresponding statistical power, and a specified significance level. Sensitivity analysis, a form of power analysis particularly useful after data collection, allows for the estimation of minimum detectable effect sizes under realistic model assumptions. We generated sensitivity curves for two key analyses: (1) whether brainprint similarity at baseline is predicted by birth weight discordance × zygosity (representing early environmental effects; see Table S1: Model 1), and (2) whether brainprint similarity is influenced by the interaction between zygosity and training status (representing late environmental effects; see Table S1: Model 5). Specifically, Sensitivity curves were created using the simr r-package (n = 1000 simulations per model), with effect sizes increasing stepwise across simulations. Model structures and covariates were based on the empirical data, and we used linear models (lm [formula 1] and lmer [formula 5]) instead of generalized additive models (GAMs) for computational simplicity. In both cases, the interaction term was the effect of interest. See Sensitivity curves in Figure S9C-D. The sensitivity analysis showed that for both tests, the results have enough power, i.e, ≥ .8, to detect effects of medium size

($\eta^2_p \approx .08$ and .05 for the early and later environmental tests), which correspond roughly to raw effects on brainprint similarity of ≈0.0175 and 0.0023.

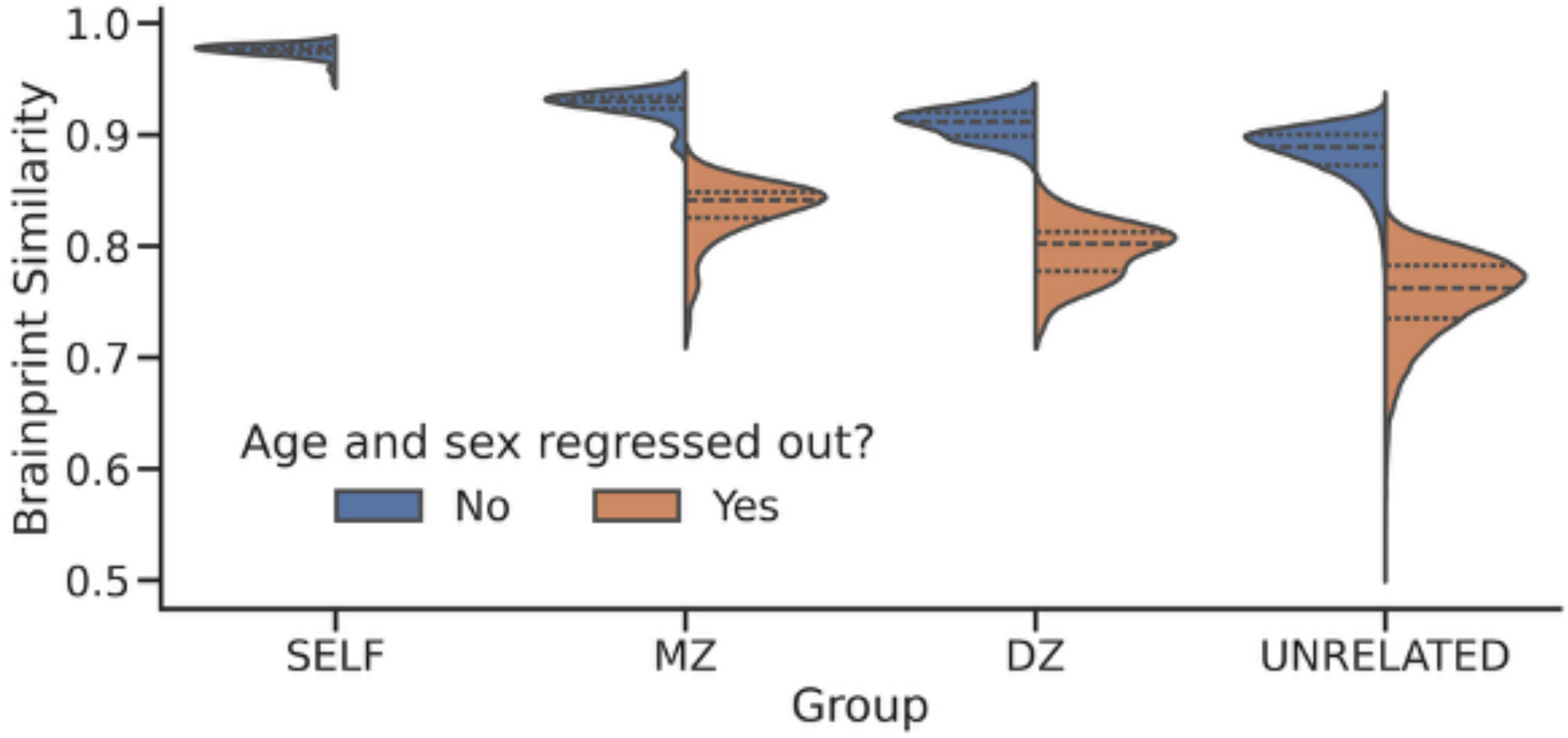

Fig. S1. Distribution of brainprint similarity scores, both with and without regressing out age and sex from brain features going into the brainprints. The self-similarity distribution is here calculated from a subgroup of **127** individuals with two MRI sessions before training (passive non-trainers or ”start_rest”-group), separated by about 10 weeks.

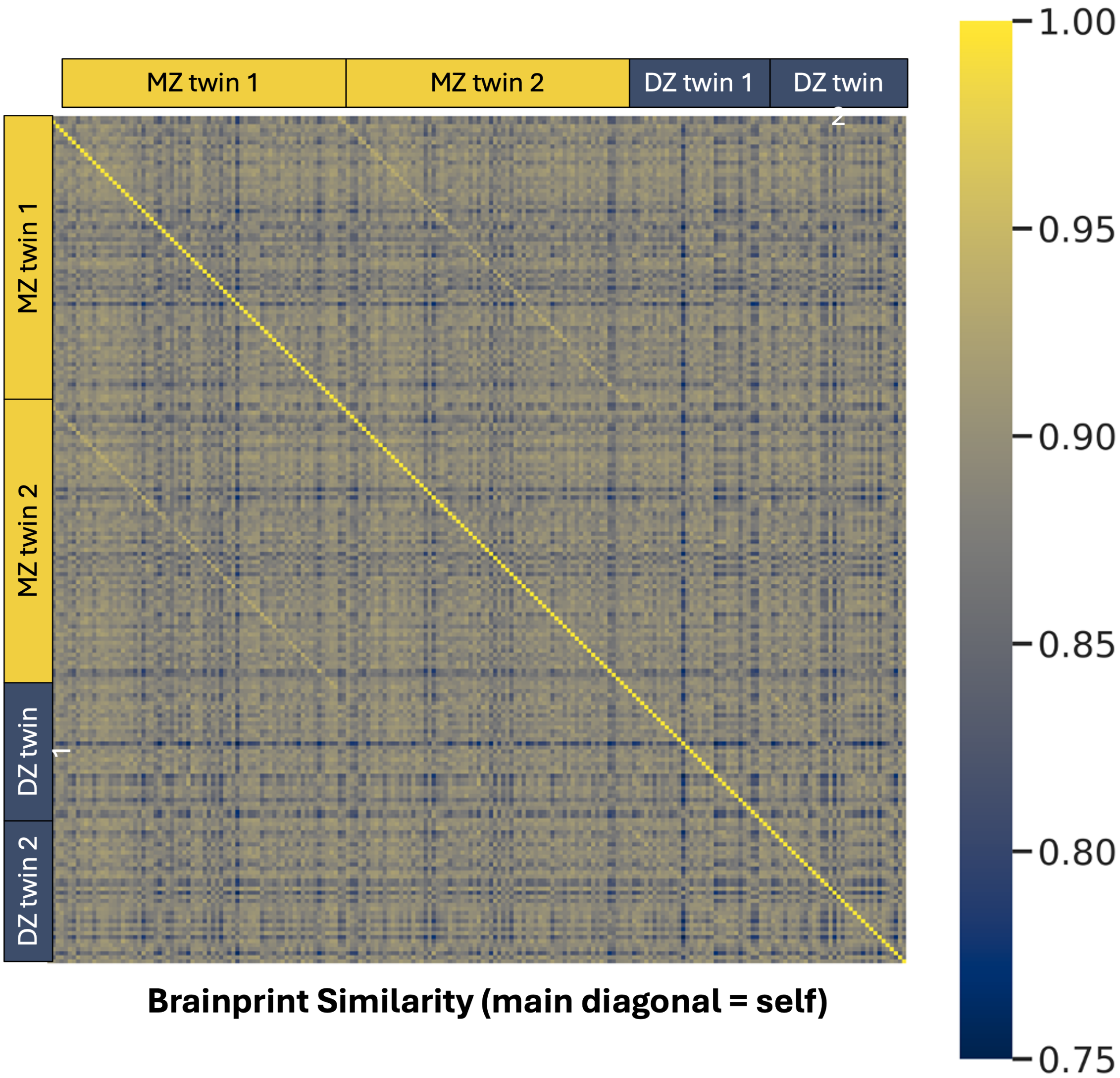

Fig. S2. Raw brainprint similiarity at baseline, based on cortical area (CA), thickness (CT), volume (CV) and mean curvature (MC). Neither age, nor sex is controlled for here (compare to Figure 1A, showing brainprint similarity based on residuals when age and sex are controlled for). N = 210, 105 twin pairs (71 MZ, 34 DZ).

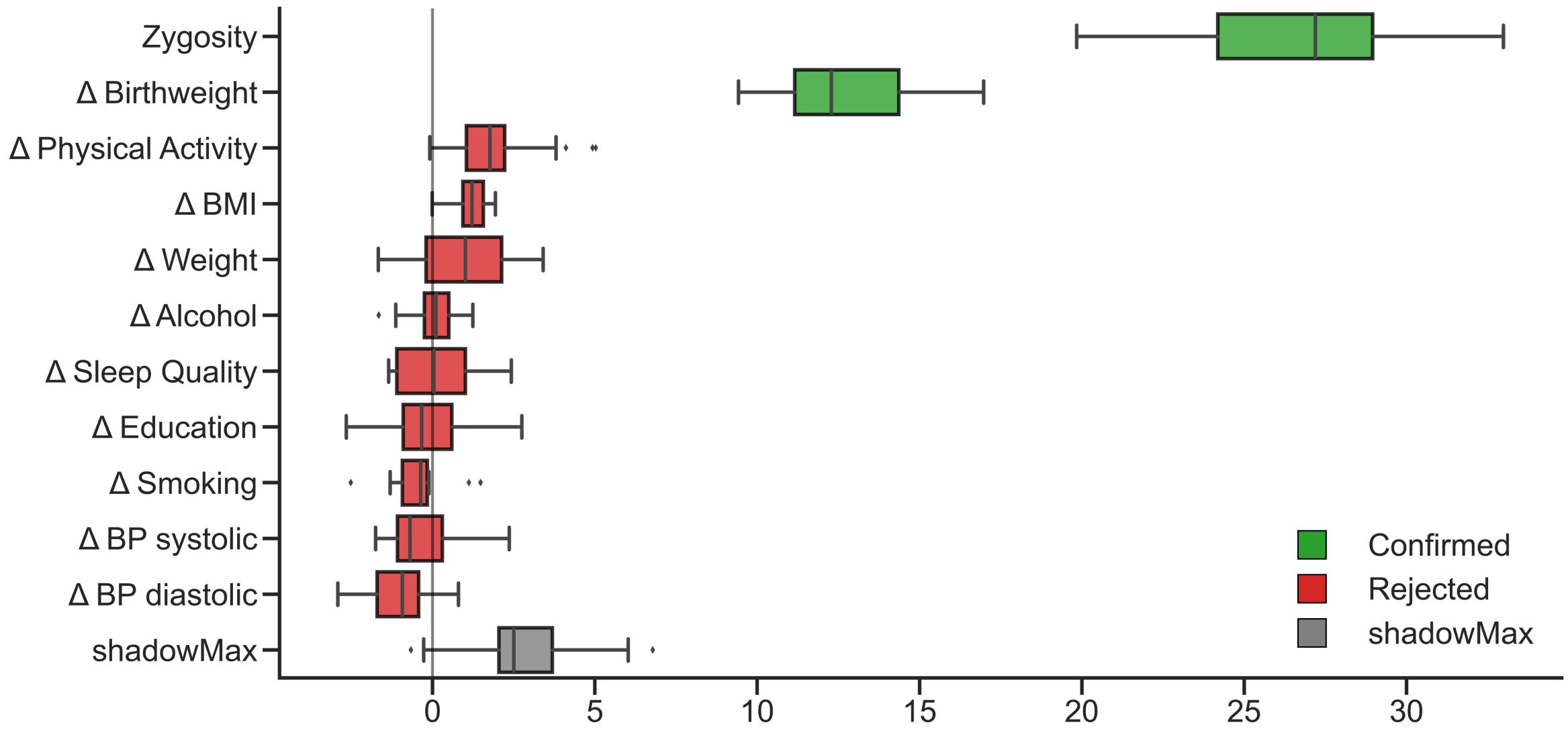

Fig. S3. Feature selection using Random Forest (Boruta) on full sample of MZ and DZ twins. Zygosity comes out as the most important feature predicting brainprint similarity within twin pairs, followed by birthweight discordance. No other environmental discordance measures reached significance.

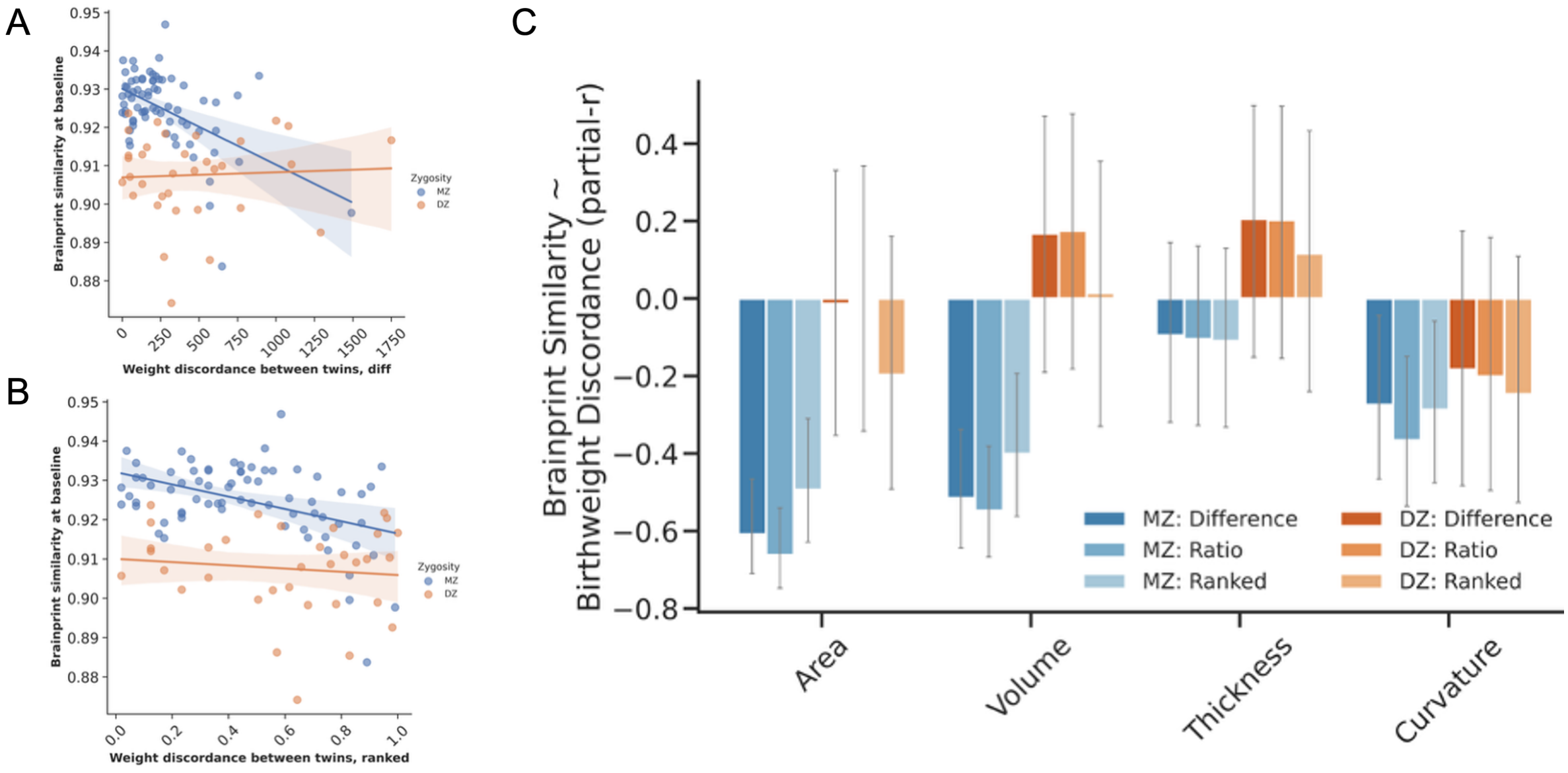

Fig. S4. Birthweight discordance ranked. A) Same plot as main Figure 2B but with birthweight discordance operationalized as difference B) Simple ranking of weight discordance differences to achieve robust statistics. GAM Model 1 on ranked data produced similar conclusions as for the non-ranked operationalizations: brainprint similarity at baseline varied as a function of zygosity ($t = -5.28$, $p < .0001$) and weight discordance ($t = -3.91$, $p = .0002$). Weight discordance was significantly correlated with brainprint similarity in MZ ($r = -.43$, $p = .0002$), but not in DZ twins ($r = -.11$, $p = .54$). The interaction of zygosity and weight discordance was however not significant for ranked data ($t = 1.706$, $p = .09$). C) Same plot as main figure 2C, with data from other BW discordance operationalizations added. Cortical area and volume effects dominated also using ranked data (both $p <= .001$; curvature $p = .018$), and only in MZ twins (all DZ $p > .10$): increased MZ birthweight discordance (ranked) was associated with lower brainprint similarity in adulthood. Errorbars indicate 95% CI.

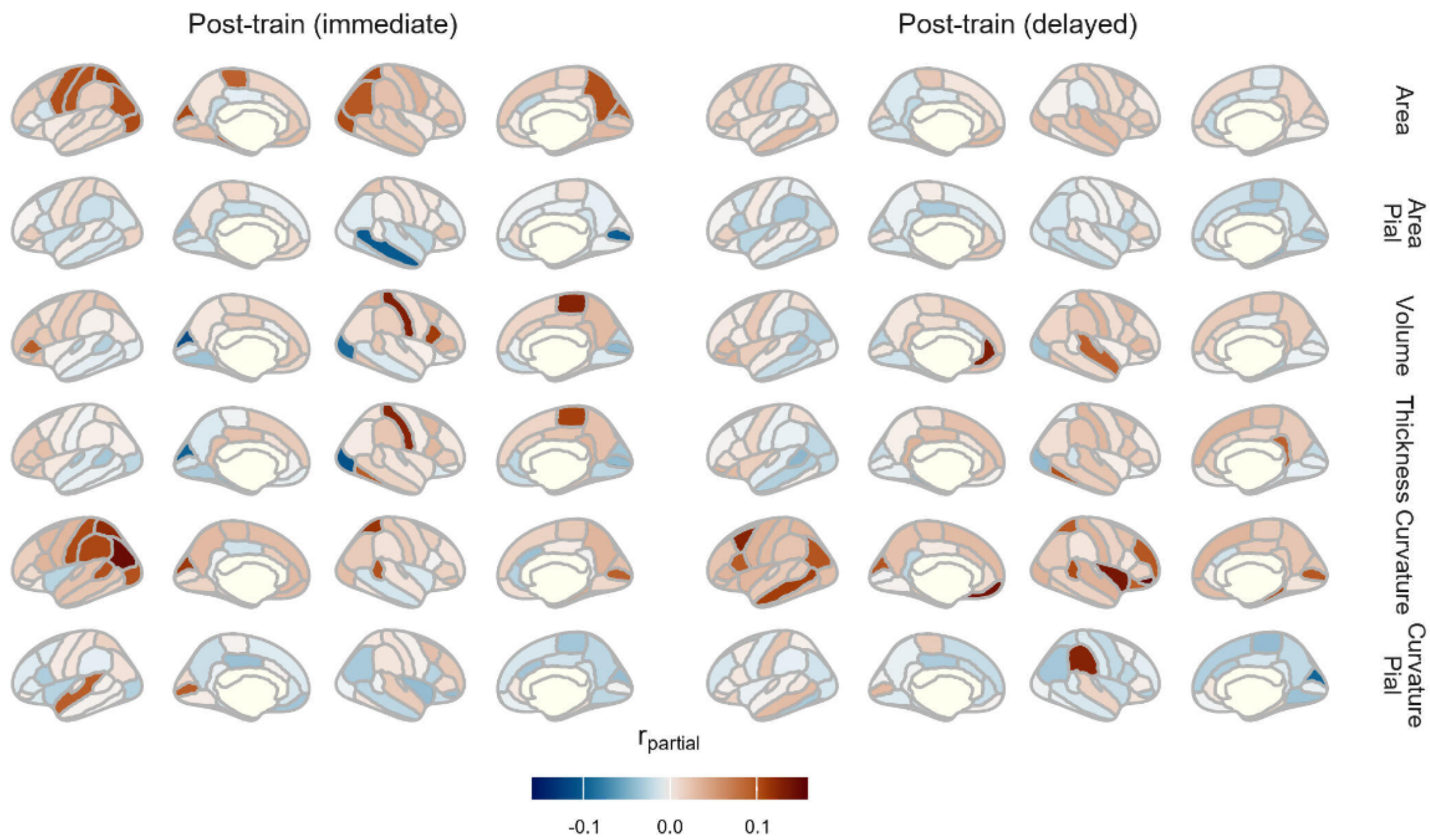

Fig S5. Effect of training status on regional cortical structure. The maps show partial correlation coefficients (r-partial) associated with training status (immediate and delayed post-train periods) and zygosity, from the GAMM model (Table S1, Model 6-7). Positive values indicate increase in regional cortical structure, i.e., relative increase in regional cortical area and curvature. Colours represent the partial correlation coefficient (r-partial) from the GAMM model, indicating the strength and direction of the association. Opaque regions are significant $p < 0.05$ (uncorrected). Note that no interpretation of these results is done at a regional level. The six rows correspond to different feature categories (Area, Area measured at the pial surface, Volume, Thickness, Curvature and Curvature measured at the pial surface). Brain views show left and right hemispheres, lateral and medial surfaces.

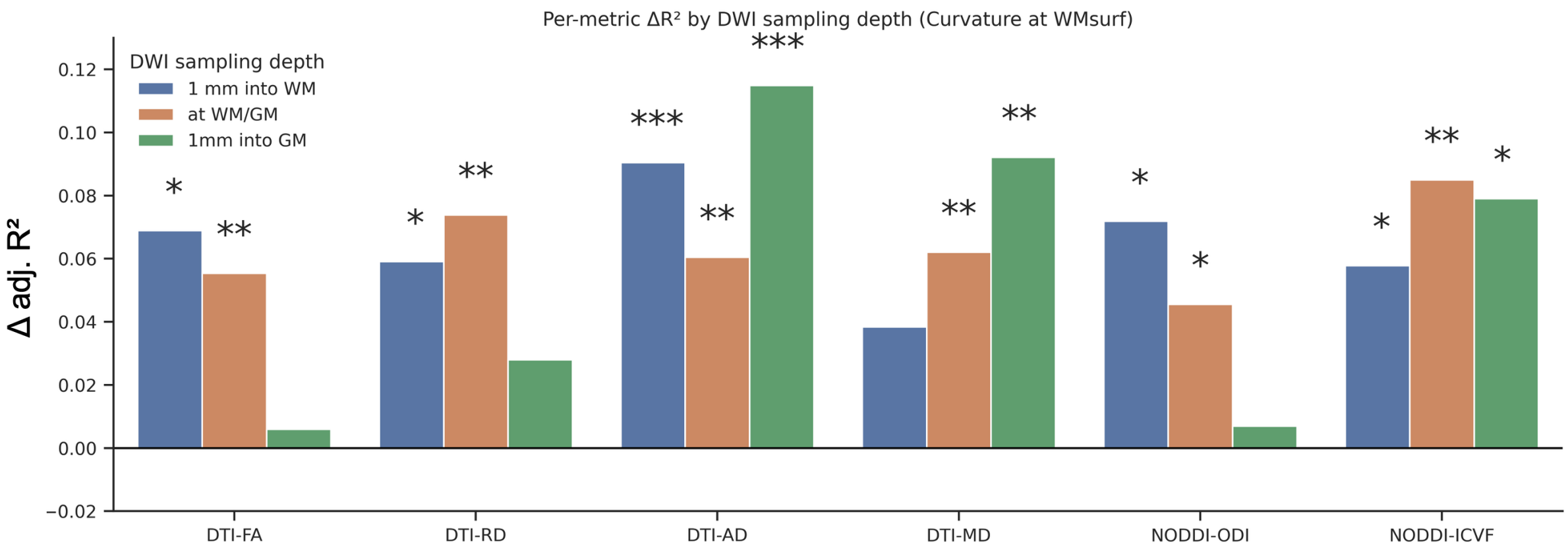

Fig. S6. Per-metric improvement in predicting WM-surface curvature similarity when adding diffusion brainprints. For each diffusion metric a global twin-similarity predictor was computed at three depths (1 mm into WM, at WM/GM, 1 mm into GM) and added with its interaction with training to the model (Table S2: Model 8). Bars show change in adjusted R^2 versus the baseline model without diffusion (Table S2: Model 5). Asterisks mark likelihood ratio test p-values after FDR correction across depths within each metric, evaluated at alpha = 0.05/6 due to six metrics (* p <.05, ** p < .01, *** p < .001).

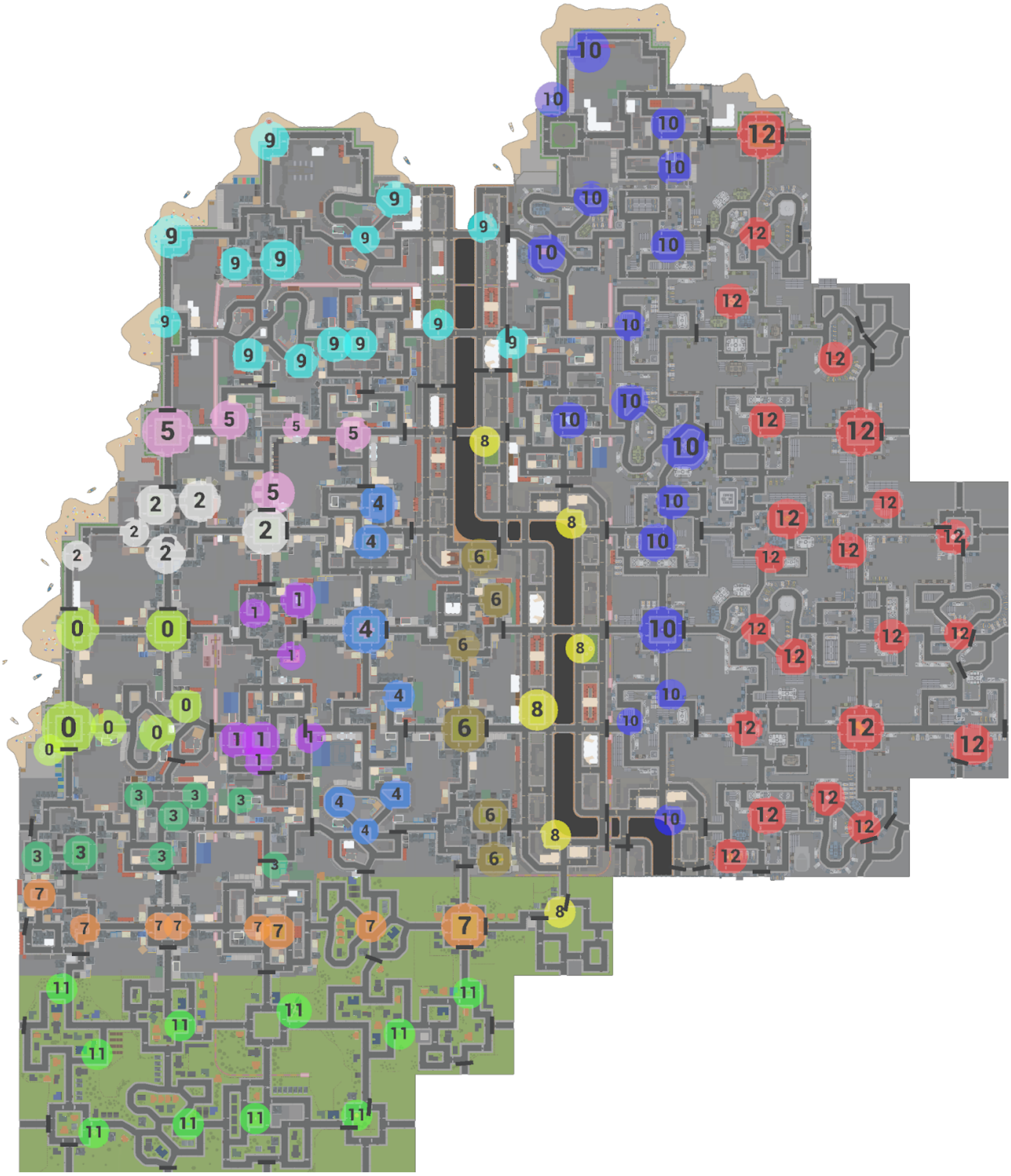

Fig. S7. Distribution of targets, color coded per level, in Plasti City

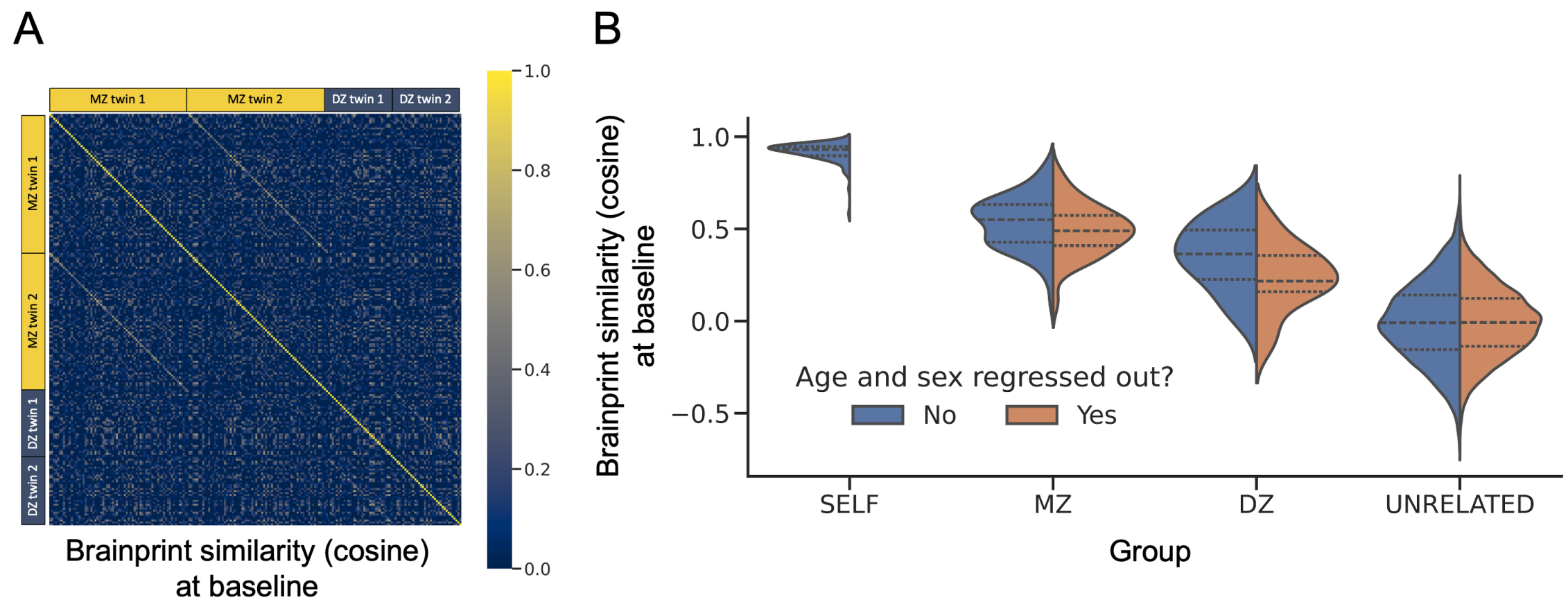

Fig. S8. Cosine similarity as metric. A) Raw brainprint similarity calculated as for Fig. S2 but with cosine similarity as alternative metric. B) See caption to Fig. S1.

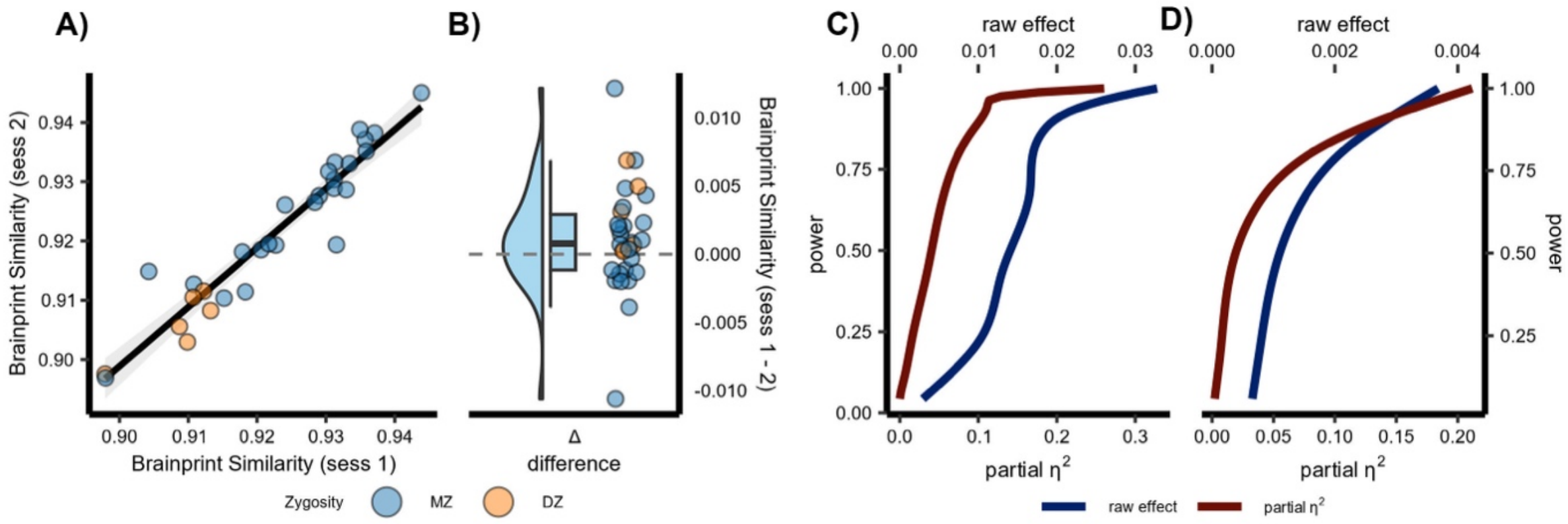

Fig. S9. Precision and Sensitivity of brainprint similarity. Psychometric properties of brainprint similarity. A) Correlation of brainprint similarity between two different passive sessions (session 1 & session 2) for 30 twins (22 MZ). Colors represent zygosity. The line represents the correlation between session 1 and session 2 and the ribbon represents its standard error. B) Within-subject differences in brainprint similarity; i.e., random variability in brainprint similarity across repeated scans. Sensitivity analysis for the main C) early (BW discordance × zygosity) and D) late (training status × zygosity) environmental effects on brainprint similarity. The plots illustrate the relationship between effect size (raw, standardized) and power in our dataset.

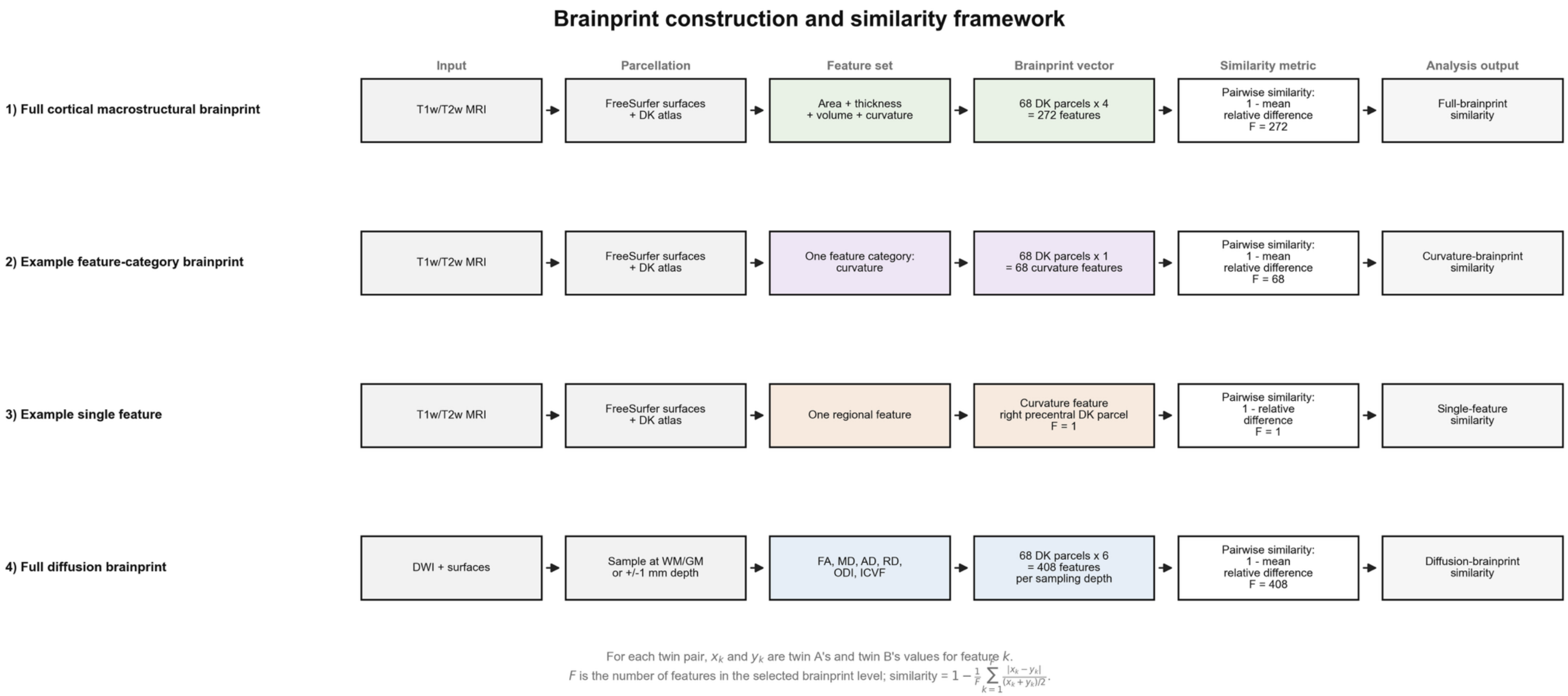

Fig. S10. Brainprint construction and similarity framework. Schematic overview of how brainprints were constructed at different levels of feature aggregation. For cortical macrostructure, regional features were extracted from FreeSurfer surfaces using the Desikan–Killiany atlas. The full cortical macrostructural brainprint comprised 272 features: 68 parcels for each of area, thickness, volume, and curvature. Feature-category brainprints used one feature class at a time, and single-feature analyses computed similarity for individual regional features. Diffusion brainprints were constructed from diffusion-derived metrics sampled relative to the gray–white matter boundary. For each twin pair, similarity was defined

as one minus the mean relative difference across the selected feature set, where F denotes the number of features included in the corresponding brainprint. The schematic illustrates the common feature-construction and similarity framework; analysis-specific covariate adjustment and sensitivity analyses, including ICV/allometric normalization, are described in the Methods and applied as specified for each analysis.

Supplementary References

1 Walhovd, K. B. *et al.* Genetic risk for Alzheimer disease predicts hippocampal volume through the human lifespan. *Neurol Genet* **6**, e506 (2020). https://doi.org:10.1212/NXG.0000000000000506

2 Chang, C. C. *et al.* Second-generation PLINK: rising to the challenge of larger and richer datasets. *Gigascience* **4**, 7 (2015). https://doi.org:10.1186/s13742-015-0047-8
