# Supplementary material for: Feature-specific environmental contributions to human cortical architecture revealed by twin brainprints
Source: Commun Biol. 2026 Jul 20;9:1503. doi: 10.1038/s42003-026-10707-w (PMC13752308; doi:10.1038/s42003-026-10707-w)
Supplement: Supplementary file 3 [file 42003_2026_10707_MOESM3_ESM.pdf]

Description of Additional Supplementary Files

File name: Supplementary Data 1

Description: Source data with numbers underlying the figures have been uploaded

and can be obtained as Supplementary Data 1. This data set has now been referred to in connection with the figures in the manuscript file.
