# Supplementary material for: Feature-specific environmental contributions to human cortical architecture revealed by twin brainprints
Source: Commun Biol. 2026 Jul 20;9:1503. doi: 10.1038/s42003-026-10707-w (PMC13752308; doi:10.1038/s42003-026-10707-w)
Supplement: Supplementary file 4 [file 42003_2026_10707_MOESM5_ESM.pdf]

nature portfolio

Corresponding author(s): Kristine B Walhovd

Last updated by author(s): 06/25/2026

Reporting Summary

Nature Portfolio wishes to improve the reproducibility of the work that we publish. This form provides structure for consistency and transparency in reporting. For further information on Nature Portfolio policies, see our Editorial Policies and the Editorial Policy Checklist.

Statistics

For all statistical analyses, confirm that the following items are present in the figure legend, table legend, main text, or Methods section.

n/a	Confirmed	
☐	☒	The exact sample size (*n*) for each experimental group/condition, given as a discrete number and unit of measurement
☐	☒	A statement on whether measurements were taken from distinct samples or whether the same sample was measured repeatedly
☐	☒	The statistical test(s) used AND whether they are one- or two-sided<br>*Only common tests should be described solely by name; describe more complex techniques in the Methods section.*
☐	☒	A description of all covariates tested
☐	☒	A description of any assumptions or corrections, such as tests of normality and adjustment for multiple comparisons
☐	☒	A full description of the statistical parameters including central tendency (e.g. means) or other basic estimates (e.g. regression coefficient) AND variation (e.g. standard deviation) or associated estimates of uncertainty (e.g. confidence intervals)
☐	☒	For null hypothesis testing, the test statistic (e.g. *F*, *t*, *r*) with confidence intervals, effect sizes, degrees of freedom and *P* value noted<br>*Give P values as exact values whenever suitable.*
☒	☐	For Bayesian analysis, information on the choice of priors and Markov chain Monte Carlo settings
☒	☐	For hierarchical and complex designs, identification of the appropriate level for tests and full reporting of outcomes
☐	☒	Estimates of effect sizes (e.g. Cohen's *d*, Pearson's *r*), indicating how they were calculated

Our web collection on statistics for biologists contains articles on many of the points above.

Software and code

Policy information about availability of computer code

Data collection	Data collection software comprised: Unreal Engine 4.22, used to build and run the VR navigation training task (Plasti City); Python with the scikit-image package, used for shortest-path performance analysis (MCP algorithm); custom web-based logging software and study laptops for online and at-home training data collection; the Illumina Global Screening Array for genotyping; PLINK2 for kinship coefficient computation and zygosity determination; and a Siemens Prisma 3T MRI scanner with Prescan Normalize enabled for neuroimaging data acquisition.
Data analysis	Data analysis software comprised: FreeSurfer v7.3.2 for structural MRI preprocessing, including cross-sectional recon-all and longitudinal -base/-long pipelines, and SAMSEG for intracranial volume estimation; QuNex container v1.0.3 implementing the HCP Diffusion Preprocessing Pipeline v5.0.0 for DWI preprocessing, including FSL TOPUP for distortion correction, FSL Eddy for motion and eddy-current correction, and FSL DTIFIT for diffusion tensor fitting (FSL version as per HCP pipeline v5.0.0); the CUDIMOST Watson model as implemented in QuNex for NODDI modelling; nilearn (surface.vol_to_surf) for sampling DWI metrics to cortical surfaces; Python 3.10.4 and pingouin 0.5.5 for general statistical computations; rpy2 3.5.16 for R–Python interfacing; R 4.2.1 with the mgcv and gamm4 packages for generalized additive models (GAMs) and generalized additive mixed models (GAMMs); R 4.4.2 with the randomForest and Boruta packages for random-forest-based feature selection; the psych R package for intraclass correlation analyses; and the simr R package for sensitivity and power analyses. Analysis code is publicly available at https://github.com/LCBC-UiO/Set-to-change.

For manuscripts utilizing custom algorithms or software that are central to the research but not yet described in published literature, software must be made available to editors and reviewers. We strongly encourage code deposition in a community repository (e.g. GitHub). See the Nature Portfolio guidelines for submitting code & software for further information.

Data

Policy information about availability of data

All manuscripts must include a data availability statement. This statement should provide the following information, where applicable:

- Accession codes, unique identifiers, or web links for publicly available datasets
- A description of any restrictions on data availability
- For clinical datasets or third party data, please ensure that the statement adheres to our policy

The LCBC dataset has restricted access, requests can be made to the corresponding author, and some of the data can be made available given appropriate ethical and data protection approvals. However, the registry data on birth weight connected to this sample are not shareable by the authors, as these data are owned by the Medical Birth Registry of Norway https://www.fhi.no/en/hn/health-registries/medical-birth-registry-of-norway/medical-birth-registry-of-norway/ , and the Norwegian Twin Registry https://www.fhi.no/en/more/health-studies/norwegian-twin-registry/ so that any access to data must be approved by them. The code used for main analyses can be accessed here https://github.com/LCBC-UiO/Set-to-change

Research involving human participants, their data, or biological material

Policy information about studies with human participants or human data. See also policy information about sex, gender (identity/presentation), and sexual orientation and race, ethnicity and racism.

Reporting on sex and gender	Biological sex was recorded at registration via the Norwegian Twin Registry and confirmed through DNA-based zygosity analysis. Gender was not separately assessed. The study design required same-sex twin pairs only (both MZ and DZ), meaning the sample includes male–male and female–female pairs but no mixed-sex pairs. This constraint is methodological: including opposite-sex DZ pairs would confound within-pair cortical comparisons with sex effects. Sex was included as a covariate in all relevant models, and age and sex were regressed from brainprint features in identification analyses. No sex-stratified analyses were performed for main analyses, as the within-pair design inherently controls for sex in all primary comparisons. Source data contain sex disaggregated at individual level. We tested whether sex influenced the birthweight-discordance association. Because all included twin pairs were same-sex pairs, sex becomes a pair-level covariate in these analyses. The sex x birthweight-discordance interaction to test whether the association differed between female-female and male-male pairs; was not significant. The subgroup sizes make a formal three-way test underpowered, but we also tested the sex x zygosity x birthweight-discordance interaction; this was likewise not significant.
Reporting on race, ethnicity, or other socially relevant groupings	Race and ethnicity were not systematically collected as variables in this study. The sample was mainly recruited through the Norwegian Twin Registry, which is population-based and draws from Norway's national birth records. The sample is therefore predominantly of Northern European ancestry. Ancestry was not used as a covariate in analyses. This constitutes a limitation of the study, as findings may not generalize beyond populations of predominantly European ancestry.
Population characteristics	The sample comprised 210 individuals from 105 same-sex twin pairs (71 MZ, 34 DZ), aged 16–78 years at baseline (mean ≈ 37 years, 72 female pairs, 33 male pairs). Participants were healthy community-dwelling volunteers recruited from the Norwegian Twin Registry and via social media. Exclusion criteria included self-reported cognitive concerns and any diagnosis known to affect CNS functioning; participants over 40 years were additionally required to score ≥25 on the MMSE. Genotypic information was available for 158 participants (DNA from saliva or buccal swabs, genotyped on the Illumina Global Screening Array), confirming registry-based zygosity classifications in all cases. No current psychiatric or neurological diagnoses were present in the sample by design. Key covariate-relevant characteristics measured at baseline are reported in Table 1 of the manuscript and include: birthweight (registry- or self-report-based), current weight, BMI, systolic and diastolic blood pressure, alcohol consumption (units/week), smoking history (never/previous/current), years of education, sleep quality (Pittsburgh Sleep Quality Index global score), and physical activity (International Physical Activity Questionnaire total score). For intervention participants, training time and navigation level reached are additionally reported in the manuscript.
Recruitment	Participants were recruited primarily through the Norwegian Twin Registry (NTR; n=190, 90.5%), selected based on availability of birth weight data for both twins from the Medical Birth Registry of Norway. A minority (n=20, 9.5%) was recruited via social media after NTR recruiting was paused due to COVID-19-related institutional burden. Only same-sex pairs were included. Participants were required to be healthy community-dwelling volunteers with no self-reported cognitive concerns and no diagnoses known to affect CNS functioning; those over 40 years were required to score ≥25 on the MMSE. Potential sources of bias: NTR selection on birth weight data availability may oversample younger pairs (registry records available from 1967); social media recruitment may introduce self-selection bias toward more health-conscious or technologically engaged individuals. These factors are unlikely to systematically bias the primary within-pair brainprint comparisons, which control for shared environmental and genetic background.
Ethics oversight	The study was approved by the Regional Committee for Medical Research Ethics, South Norway. All participants provided written informed consent prior to participation. Birth weight data were obtained from the Medical Birth Registry of Norway and the Norwegian Twin Registry under appropriate ethical and data protection approvals governed by the Norwegian Institute of Public Health.

Note that full information on the approval of the study protocol must also be provided in the manuscript.

Field-specific reporting

Please select the one below that is the best fit for your research. If you are not sure, read the appropriate sections before making your selection.

☒ Life sciences ☐ Behavioural & social sciences ☐ Ecological, evolutionary & environmental sciences

For a reference copy of the document with all sections, see nature.com/documents/nr-reporting-summary-flat.pdf

Life sciences study design

All studies must disclose on these points even when the disclosure is negative.

Sample size

The study used an existing twin sample (Set-to-change study) with 105 twin pairs (71 MZ, 34 DZ; 210 individuals, age 16–78 years). No a priori power calculation was conducted for the observational brainprint analyses. Post-hoc sensitivity analyses (simr package, 1000 simulations per model) confirmed adequate power (≥0.80) to detect medium-sized effects ($\eta^2p \approx 0.08$ for the early environment × zygosity interaction; $\eta^2p \approx 0.05$ for the training × zygosity interaction).

Data exclusions

Four MRI datasets from wave 1 were excluded due to scan artefacts (n=2) or human data loss (n=2); these participants contributed data at waves 2–3. One participant was excluded following a clinical radiological finding. Eleven participants had valid MRI data for only one twin in the pair and were excluded from pair-wise analyses. Two twin pairs (1 MZ, 1 DZ) were excluded from DWI analyses due to missing DWI data for at least one twin. Nine twin pairs initially enrolled in training were reclassified as passive non-trainers (did not meet minimum engagement: ≥2 hours training and progression beyond baseline navigation level).

Replication

Within-study replication: birthweight discordance effects on brainprint similarity were tested with multiple operationalizations of BW discordance (continuous difference in grams, ratio score, rank-transformed; Figures 2B and S4) and yielded convergent conclusions. Brainprint similarity showed excellent test-retest reliability (ICC(2,1) = 0.947; n=30 twin pairs with two passive scans). No independent external replication cohort was used.

Randomization

For the intervention, twins were randomly assigned to start with either a training (A) or rest (B) condition. After ~10 weeks, repeated scans and cognitive assessments were undertaken, and participants were to switch conditions (A-B/B-A). After another ~10 weeks, scans and cognitive assessments were again repeated (see Table 2). Nine twin pairs initially enrolled in training were reclassified as passive non-trainers (did not meet minimum engagement: ≥2 hours training and progression beyond baseline navigation level).

Blinding

A radiologist evaluated clinical MRI scans blinded to group allocation for clinical purposes. For the primary brainprint statistical analyses, investigators were not formally blinded to zygosity or training condition, as these are necessary covariates in the models. The brainprint metric itself is a quantitative, automated measure not subject to rater subjectivity.

Reporting for specific materials, systems and methods

We require information from authors about some types of materials, experimental systems and methods used in many studies. Here, indicate whether each material, system or method listed is relevant to your study. If you are not sure if a list item applies to your research, read the appropriate section before selecting a response.

Materials & experimental systems

n/a	Involved in the study	
☒	☐	Antibodies
☒	☐	Eukaryotic cell lines
☒	☐	Palaeontology and archaeology
☒	☐	Animals and other organisms
☒	☐	Clinical data
☒	☐	Dual use research of concern
☒	☐	Plants

Methods

n/a	Involved in the study	
☒	☐	ChIP-seq
☒	☐	Flow cytometry
☐	☒	MRI-based neuroimaging

Plants

Seed stocks

Report on the source of all seed stocks or other plant material used. If applicable, state the seed stock centre and catalogue number. If plant specimens were collected from the field, describe the collection location, date and sampling procedures.

Novel plant genotypes

Describe the methods by which all novel plant genotypes were produced. This includes those generated by transgenic approaches, gene editing, chemical/radiation-based mutagenesis and hybridization. For transgenic lines, describe the transformation method, the number of independent lines analyzed and the generation upon which experiments were performed. For gene-edited lines, describe the editor used, the endogenous sequence targeted for editing, the targeting guide RNA sequence (if applicable) and how the editor was applied.

Authentication

Describe any authentication procedures for each seed stock used or novel genotype generated. Describe any experiments used to assess the effect of a mutation and, where applicable, how potential secondary effects (e.g. second site T-DNA insertions, mosiacism, off-target gene editing) were examined.

Magnetic resonance imaging

Experimental design

Design type

Longitudinal T1w and diffusion weighted imaging. Longitudinal structural MRI study with up to three sessions per participant (~10-week intervals), embedded in a crossover navigation training intervention. Scans were acquired at wave 1 (baseline), wave 2 (immediately post-training or post-rest), and wave 3 (post-training/delayed follow-up). Key between-subject variables: zygosity (MZ/DZ), training vs. passive non-trainer status. Key within-subject variable: training status (pre-training / immediately post-training / delayed post-training). No task was performed during MRI acquisition (all structural sequences). Total MRI observations: 488.

Design specifications

3 Tesla Siemens Prisma scanner with 32-channel head coil, at Rikshospitalet, Oslo University Hospital. All sequences acquired with Prescan Normalize enabled. Three sequences contributed to brainprint analyses:T1w MPRAGE: 208 sagittal slices, TR=2400 ms, TE=2.22 ms, TI=1000 ms, flip angle=8°, voxel size=0.8×0.8×0.8 mm, FOV=240×256 mm, matrix=300×320×208, iPAT=2.T2w SPACE: 208 sagittal slices, TR=3200 ms, TE=5.63 ms, voxel size=0.8×0.8×0.8 mm, FOV=256×240 mm, matrix=320×300×208, iPAT=2.DWI (multi-band EPI): Two shells (b=1500 and 3000 s/mm², 92–93 directions + 6 b=0 per shell), TR=3230 ms, TE=89.2 ms, flip angle=78°, voxel size=1.5 mm isotropic, 92 axial slices, multiband factor=4, phase partial Fourier=6/8, PE=AP; additional b=0 PA volumes for distortion correction. Sequences follow HCP Lifespan phase 1b pilot parameters.

Behavioral performance measures

N/A, Not during scan

Acquisition

Imaging type(s)

structural, diffusion

Field strength

3T

Sequence & imaging parameters

T1w MPRAGE: 208 sagittal slices, TR=2400 ms, TE=2.22 ms, TI=1000 ms, flip angle=8°, voxel size=0.8×0.8×0.8 mm, FOV=240×256 mm, matrix=300×320×208, iPAT=2.T2w SPACE: 208 sagittal slices, TR=3200 ms, TE=5.63 ms, voxel size=0.8×0.8×0.8 mm, FOV=256×240 mm, matrix=320×300×208, iPAT=2.DWI (multi-band EPI): Two shells (b=1500 and 3000 s/mm², 92–93 directions + 6 b=0 per shell), TR=3230 ms, TE=89.2 ms, flip angle=78°, voxel size=1.5 mm isotropic, 92 axial slices, multiband factor=4, phase partial Fourier=6/8, PE=AP; additional b=0 PA volumes for distortion correction. Sequences follow HCP Lifespan phase 1b pilot parameters.

Area of acquisition

Whole brain for all sequences (T1w, T2w, DWI).

Diffusion MRI ☒ Used ☐ Not used

Parameters

Two shells (b=1500 and 3000 s/mm², 92–93 directions + 6 b=0 per shell), TR=3230 ms, TE=89.2 ms, flip angle=78°, voxel size=1.5 mm isotropic, 92 axial slices, multiband factor=4, phase partial Fourier=6/8, PE=AP; additional b=0 PA volumes for distortion correction. Sequences follow HCP Lifespan phase 1b pilot parameters.

Preprocessing

Preprocessing software

Structural (T1w/T2w): FreeSurfer v7.3.2. Each session processed cross-sectionally via recon-all with --t2pial and --hires flags (0.8 mm native resolution). Expert options applied including PlaceMMPialSurf fix. Longitudinal processing via FreeSurfer recon-all -base (participant-specific template) and recon-all -long. Training participants' templates constructed from pre- and post-intervention sessions to prevent systematic bias. Intracranial volume estimated using SAMSEG.DWI: QuNex container v1.0.3 implementing HCP Diffusion Preprocessing Pipeline v5.0.0. Steps included gradient-nonlinearity correction, susceptibility distortion correction (TOPUP), eddy-current and motion correction (FSL Eddy with outlier detection flagging >4 SD slices), and slice-outlier replacement. NODDI modelling: CUDIMOST Watson model (QuNex). Diffusion tensors: FSL DTIFIT (OLS, all non-zero b-values). DWI metrics sampled to cortical surfaces using nilearn (surface.vol_to_surf) at WM/GM interface and ±1 mm.

Normalization

No normalization to a common volumetric standard space (e.g. MNI152) was performed, as the brainprint approach operates

Normalization

entirely in each participant's native cortical space. Regional morphological measures (area, thickness, volume, curvature) were extracted per participant in native space using the Desikan-Killiany parcellation applied via FreeSurfer's surface-based spherical registration to the fsaverage template (nonlinear, surface-based); this registration was used solely for parcellation labelling and not for the brainprint analyses themselves.For longitudinal processing, a participant-specific within-subject base template was constructed using FreeSurfer's recon-all -base command (nonlinear, within-subject), and each cross-sectional session was subsequently aligned to this template via recon-all -long to ensure consistent surface placement across timepoints. For training participants, the template was constructed from sessions immediately before and after the intervention to avoid systematic bias toward either phase. In the main analyses, area and volume features were not normalized for intracranial volume (ICV) before brainprint calculation, but an ICV-normalized sensitivity analysis is also reported. For diffusion data, the HCP T1w image for each session was registered to the participant's FreeSurfer longitudinal space using boundary-based registration (linear), and the resulting transform was inverted to bring FreeSurfer surfaces into HCP T1w space for DWI metric sampling. No nonlinear registration to a standard diffusion template was applied. All inferences were based on within-subject and within-pair similarity metrics and did not depend on cross-participant spatial correspondence beyond the parcellation level.

Normalization template

No volumetric normalization template was used. Parcellation labels were assigned via FreeSurfer's built-in spherical registration to the fsaverage surface template (FreeSurfer v7.3.2). Within-subject longitudinal alignment used participant-specific base templates constructed with FreeSurfer's recon-all -base command. These templates are individual-specific and not based on any group-level or standard-space reference brain.

Noise and artifact removal

For structural MRI, FreeSurfer's longitudinal pipeline (recon-all -long) with the --t2pial and --hires flags was used; pial surface placement was refined using the auxiliary T2-weighted image to reduce dural and vessel inclusion artifacts. Expert options were applied to address known pial surface misplacements (PlaceMMPialSurf fix) and inferior frontal surface placement near the putamen (WhitePreAparc --rip-bg-no-annot). All scans were visually reviewed and four datasets were excluded due to scan artifacts. A radiologist evaluated all scans for clinical findings.For DWI, noise and artifact removal was performed within the HCP Diffusion Preprocessing Pipeline v5.0.0 as implemented in QuNex v1.0.3. This included gradient-nonlinearity correction, susceptibility distortion correction via FSL TOPUP, and eddy-current and motion correction via FSL Eddy with outlier detection and replacement enabled, flagging and replacing slices exceeding 4 standard deviations from the expected signal. Multiband slice-group structure was specified to ensure correct outlier replacement for simultaneously acquired slices.

Volume censoring

Volume censoring was not applied. This study used structural MRI (T1w, T2w) and diffusion-weighted MRI only; no functional or BOLD data were analysed. Slice-level outlier detection and replacement was performed during DWI preprocessing via FSL Eddy (slices exceeding 4 standard deviations flagged and replaced), but no volume-level censoring or scrubbing was performed.

Statistical modeling & inference

Model type and settings

Primary analyses used generalized additive models (GAMs) and generalized additive mixed models (GAMMs) fitted in R 4.2.1 (mgcv, gamm4 packages). Age was modelled as a smooth spline term (s(age)). Twin pair was included as a random effect in longitudinal models. All tests two-sided, α=0.05. For feature-wise regional analyses (n=19 significant features), FDR correction was applied across the 272 cortical features tested. Permutation-based p-values (5,000 permutations) were used for brainprint identification accuracy. Feature selection used the Boruta algorithm (R 4.4.2, randomForest package). Post-hoc sensitivity analyses used simr (1,000 simulations). Statistical analyses outside R used Python 3.10.4 and pingouin 0.5.5. Analysis code is available at https://github.com/LCBC-UiO/Set-to-change.

Effect(s) tested

No task-based or stimulus-based contrasts were tested, as this study used structural MRI and diffusion MRI only. The primary effects tested were: (1) the interaction between birthweight discordance and zygosity (MZ vs. DZ) on baseline brainprint similarity, testing whether prenatal environmental differences have feature-specific effects on cortical architecture that are visible only against a genetically identical background; (2) the main effect of zygosity on baseline brainprint similarity, indexing the degree to which genetic similarity is reflected in cortical structural similarity; (3) the interaction between training status (pre-training / immediately post-training / delayed post-training) and zygosity on longitudinal brainprint similarity, testing whether a controlled adult environmental intervention differentially affects cortical similarity in genetically identical versus fraternal twin pairs; and (4) the interaction between diffusion-based microstructural similarity and training status, testing whether training-related changes in macrostructural brainprint similarity are accompanied by corresponding changes at the gray-white interface. All models used generalized additive (mixed) model frameworks (GAM/GAMM) with continuous and smooth terms rather than factorial ANOVA designs. Age was modelled as a nonlinear smooth spline term throughout. All tests were two-sided with α = 0.05. FDR correction was applied for regional feature-wise follow-up analyses across 272 cortical features. Permutation-based p-values (5,000 permutations) were used for brainprint identification accuracy.

Specify type of analysis: ☐ Whole brain ☐ ROI-based ☒ Both

Anatomical location(s)

No a priori regions of interest were defined. All primary analyses were conducted at the whole-brain level using brainprint similarity as a single composite metric aggregating 272 cortical features (68 regions × 4 morphological measures: area, thickness, volume, and curvature), derived from the Desikan-Killiany (DK) atlas as implemented in FreeSurfer v7.3.2. The DK atlas parcellation was applied via FreeSurfer's standard surface-based spherical registration and defines 68 cortical regions per hemisphere based on sulcal and gyral anatomy.Regional follow-up analyses were conducted in a data-driven, hypothesis-free manner: feature-wise associations between birthweight discordance and individual brainprint features were tested across all 272 features simultaneously, with FDR correction applied to control for multiple comparisons. Nineteen features survived FDR correction and are reported in Figure 2D; these were not selected a priori but emerged from the data-driven feature-wise analysis. Similarly, regional cortical maps for training effects (Figure S5) were exploratory and are explicitly presented as uncorrected, with the caveat stated in the manuscript that no regional-level interpretation was made. Anatomical localization of significant features was therefore entirely atlas-based (DK parcellation) rather than determined by independent localiser scans, functional constraints, or manual tracing.

Statistic type for inference

(See Eklund et al. 2016)

No voxel-wise or cluster-wise inference was performed. All primary analyses operated on parcellated regional summary statistics derived from the Desikan-Killiany atlas (68 regions per morphological measure), not on individual voxels or surface vertices. Brainprint similarity was computed as a single composite metric across all 272 parcellated features, and statistical inference was performed at this whole-brainprint level using generalized additive (mixed) models.For regional follow-up analyses, inference was conducted at the parcel level (one value per DK region per morphological measure per participant), with FDR correction applied across the 272 features tested simultaneously. No spatial contiguity or cluster extent thresholding was applied, as the unit of analysis was the atlas parcel rather than individual vertices or voxels. Exploratory regional maps shown in Figure S5 display uncorrected partial correlation coefficients at the vertex level for illustrative purposes only; no formal vertex-wise or cluster-wise inference was performed on these maps, and the manuscript explicitly states that no regional-level interpretation is made from them.

Correction

Three correction approaches were used depending on the analysis. For regional feature-wise analyses testing associations between birthweight discordance and individual brainprint features across all 272 cortical features (68 regions × 4 morphological measures), false discovery rate (FDR) correction was applied, with 19 features surviving correction. For brainprint identification accuracy (self-identification and twin co-twin identification), significance was evaluated using permutation-based null distributions (5,000 permutations), which inherently account for multiple comparisons by deriving empirical p-values directly from the data. For the primary hypothesis tests on composite brainprint similarity (birthweight discordance × zygosity interaction; training status × zygosity interaction), no correction for multiple comparisons was applied, as these represented a small number of pre-specified hypotheses tested in dedicated models. Exploratory regional maps (Figure S5) are presented uncorrected and are not interpreted at the regional level. No corrections for multiple comparisons across the full set of models reported in Tables S1 and S2 were applied, as these include both primary and follow-up sensitivity analyses that were not treated as independent hypothesis tests.

Models & analysis

n/a	Involved in the study
☒	☐ Functional and/or effective connectivity
☒	☐ Graph analysis
☒	☐ Multivariate modeling or predictive analysis
